# Healthcare workers’ perspectives on healthcare-associated infections and infection control practices: a video-reflexive ethnography study in the Asir region of Saudi Arabia

**DOI:** 10.1186/s13756-020-00756-z

**Published:** 2020-07-16

**Authors:** Esther Paul, Ibrahim A. Alzaydani Asiri, Ahmed Al-Hakami, Harish C. Chandramoorthy, Sarah Alshehri, C. M. Beynon, Abdullah M. Alkahtani, Ali H. Asiri

**Affiliations:** 1 Department of Microbiology and Clinical Parasitology and Stem cell unit, College of Medicine, King Khalid University, PO Box: 641, Abha, 61421 Saudi Arabia; 2 Department of Paediatrics, Maternity and Children’s Hospital, Ministry of Health, 62521 Emirate Al Shifa, Abha, Saudi Arabia; 3 Department of Otolaryngology, Head & Neck Surgery, College of Medicine, King Khalid University, PO. Box: 641, Abha, 61421 Saudi Arabia; 4 Honorary Lecturer, Laureate International Universities, Abha, Saudi Arabia

**Keywords:** Healthcare worker, Infection control, Saudi Arabia, Healthcare-associated infection

## Abstract

**Background:**

Healthcare-associated infections (HAIs) are a global public health problem. For the fulfillment of Saudi Arabia’s Vision 2030, the promotion of preventive care medicine through HAI management is a crucial issue. This study explores the perspectives of Saudi tertiary healthcare workers (HCWs) on HAIs and infection control measures.

**Methods:**

Quantitative data were assessed to determine HCWs’ knowledge of HAI and their attitudes towards and practice of infection control measures. Semi-structured interviews were used to collect qualitative data from 40 doctors and nurses. The interviews were audio-recorded and transcribed verbatim. Further, routine sterile procedures in the wards and intensive care units were video recorded, and the footage was discussed by the infection control team and the personnel involved in the videos. This discussion was videographed and transcribed. Both interview data and reflective discussion of the video were analysed using thematic analysis. The quantitative data were analysed using the Kruskal–Wallis test and logistic regression analysis.

**Results:**

Kruskal–Wallis test revealed no difference in mean knowledge, attitude, or practice scores between nurses/ doctors or the genders. There was a significant difference in knowledge score and practice scores between the Intensive care unit & the Paediatric ward /infection control department with the maximum scores in knowledge and practice among participants from the intensive care unit. Logistic regression analysis for dependent variables (knowledge and attitude) and independent variables like age, gender, designation, and departments was not significant. The qualitative data yielded four themes: knowledge of HAI and infection control, infection control measures in practice, a shortfall in infection control measures and HAI, and required implementation. Video-reflexive ethnography (VRE) revealed lapses in handwashing practice and proper usage of personal protective equipment (PPE), especially surgical masks.

**Conclusion:**

Early introduction of training programmes in medical and nursing schools and video demonstrations of appropriate infection control practices during sterile procedures would be highly beneficial to HCWs. A possible reason for the outbreak of Middle East Respiratory Syndrome coronavirus in this part of Saudi Arabia could be a lapse in PPE usage. Intensive training programs for all the HCWs, strict vigilant protocols, and a willingness to change behaviour and practice, will significantly benefit the spread of outbreaks.

## Background

Globally, Healthcare-associated infections (HAIs) are a serious public health problem. They are the primary cause of the increase in morbidity and mortality of patients and involve high costs, increased need for advanced medical care, the use of expensive antibiotics to treat HAIs, which may lead to the emergence of other antimicrobial-resistant organisms [1, 2]. Healthcare workers (HCWs) (e.g., physicians, nursing personnel) often serve as vectors for HAIs; thus, their role requires serious consideration in the implementation of infection control strategies to prevent HAIs [3].

Saudi Arabia is undergoing a significant change as it marches towards the fulfillment of its ambitious ‘Vision 2030’. In this context, an essential aim of the national ministry of health is to promote preventive care medicine to tackle crucial issues like HAIs, implement adequate infection control measures, and prevent antibiotic resistance [4].

Over a span of two decades, a substantial number of studies on HAIs has been published across Saudi Arabia. However, most are prevalence and epidemiological studies on the different types of HAIs, especially device-associated HAI or studies on the microbiological profile of various HAIs [5–7]. Studies from Riyadh and Abha have reported a prevalence of HAI to be between 8 and 8.5% [8, 9]. Some studies in Saudi Arabia, however, have focused on HCWs’ knowledge of HAI, attitude, and practice of infection control measures; one study identified a gap in knowledge but failed to analyse in-depth the reason for it [10]. In addition, several studies have focused on the knowledge and practice of hand hygiene and ignored other aspects of infection control [11, 12]. Our study has aimed to analyse the knowledge, attitude towards HAI and practice of infection control measures in a more detailed method and has strived to study the perspectives of HCW using the qualitative Analysis and Video reflexive ethnography (VRE). The reason for choosing the Maternity and paediatric hospital for conducting this study is the report of the high periodic prevalence of 19.2% and an incidence of 13.7 HAI infections per 1000 patient days in recent years [13].

The Gulf Cooperation Council (GCC) Centre for Infection Control was established to record the rate, trends, associated risks of HAIs, and enforce methods and strategies to reduce HAIs [14, 15]. However, it is unclear if infection control protocols, as instituted by this centre, are being followed in all tertiary care hospitals in GCC countries. An ongoing problem faced by the Kingdom since 2012 is outbreaks of Middle East Respiratory Syndrome coronavirus (MERS-CoV) infections and now the recent outbreak of COVID-19 all over the world. Strict infection policies and effective use of personal protective equipment (PPE) can prevent the spread of MERS-CoV and COVID-19 [16]. All HCWs must use standard precautions related to droplet, contact, and airborne infection to contain and curb the spread of HAIs [17]. Our study highlights lapses in infection control practices, especially in the use of PPE., and makes us to speculate if these deficiencies can enhance the spread of airborne endemic infections like MERS-CoV and pandemic infections like COVID-19.

## Methods

### Study design and settings

This study was conducted over 18 months (January 2018–June 2019) in three phases at a tertiary care hospital in Saudi Arabia using a mixed methodology method. The overall aims were to explore HCWs’ perspectives on HAI and to identify current problems in infection control practices and possible solutions to them using a descriptive analysis method (Knowledge, Attitude and Practice (KAP) survey, qualitative methodology, and VRE.

### Inclusion criteria

All the HCWs who had been working in the hospital for six months or more were eligible to take part in the study.

### Exclusion criteria

All the HCWs with less than six months of work experience in the hospital were not included in the study.

### First phase

In the first phase of the study, simple random sampling was employed to select 50 participants. The total of the consultants, residents, and nurses (1066) were listed out and assigned numbers, and the participants were chosen randomly. With a margin error of 5% for a population size of 1066, the ideal sample size was 283. However, after random selection, those willing to participate in the study were 50 in number. A questionnaire developed by the authors was used to assess the KAP of HAI and infection control. The questionnaire was developed and validated based on previous studies [18, 19]. For further validation, a pilot study with 30 participants was conducted using the questionnaire, and these 30 participants were not included in the study. The final questionnaire was refined and developed as per the needs of the study and consisted of nineteen knowledge questions, ten attitude questions, and eight practice questions (Additional file 1). The participants were scored out of a total score of 37. All the positive answers under the three sections were given a score of 1. The negative answers were given a score of 0, except for a few questions. For example, the participants were scored as one if they had attended more than one workshop and 0 if they had participated in none. Similarly, the participants were given a scoring of 1 and 0 as per the preferences they chose in the questionnaire.

### Statistical analysis

The Kruskal–Wallis test, a non-parametric test, was used to determine the statistical significance of the quantitative data. We used this test to compare the scores from two or more groups of independent observations [20]. *P*-values < 0.05 were considered significant. Knowledge, practice, and attitudes were the dependent variables, and the designation, departments of the participant, age, and gender were the independent variables. However, the Kruskal–Wallis test can only determine if there is a significant difference between groups but cannot decide which group is different. Logistic regression was performed to determine the association between independent variables and dependent variables [21].

### Second phase

In the second phase of the study, we collected qualitative data via semi-structured interviews from 40 participants recruited through purposive sampling to generate useful and information-rich data [22]. The Snowball sampling technique (a multi-step purposive sampling) was used to add more people to the sample at each step. The initial phase involved the identification of the interested health care workers who further identified more healthcare workers. This technique was used to narrow down on variation and focus on similarities [23]. Semi-structured interviews were used to gain an understanding of how HCWs interpret HAI and examine their experience in tackling HAI and associated infection control measures. We followed an interview guide (Additional file 2) comprising of open-ended questions on existing HAI and infection control practices/guidelines in the healthcare facility, as well as HCWs’ awareness of HAI and the need for safe infection control measures. Interviews were audio-recorded and transcribed verbatim immediately after obtaining written informed consent from the participants. Thematic analysis was then used to extract essential themes and patterns [24]. The transcripts were coded manually; we organized codes into categories and constantly revised themes, categories, and codes as we progressed through the data analysis. The codes were used to organize, analyse, and examine the data in a structured way. A codebook was compiled from the final data to help us revisit, re-examine, and understand the codes and thus highlight and operationalize our findings.

### Third phase

The third phase of the study employed VRE, which is commonly used to identify and reflect on healthcare practitioners’ mistakes and better implement infection control practices [25]. VRE was not conducted in a covert manner as we aimed for the researchers and the participants to collaborate to provide an ethical approach to video ethnography. We tried to prevent the Hawthorne effect by disclosing sparse details about the purpose of the video recording [26]. The participants recruited for VRE were the same ones who had participated in the qualitative study. An entire routine sterile procedure performed by five teams of three or more participants in the intensive care unit (ICU) setup was recorded right from pre-procedure hand hygiene measures until its conclusion. The footage was then shown to a focus group consisting of two paediatricians, an infection control nurse, and the principal researcher. Lapses in infection control practice were identified, and solutions discussed, and the edited video footage was presented to the participants featured in the video recordings. Feedback sessions were conducted with the participants who had performed the sterile procedures and featured in the videos. Transcripts and video data were analysed, and coding was performed to identify common patterns and themes.

Debriefing and reflexivity were achieved by the maintenance of a written account throughout the research process to improve the credibility of the study [27]. The use of a digital recorder and sharing the pilot interviews with the research advisor for review ensured reliability and dependability. Sharing the pilot interviews with the research advisor helped in critiquing the research process and challenge any assumptions [28].

## Results

The demographic characteristics of the 50 participants in the quantitative study method are as follows. 48% were between 20 and 29 years of age, 36% were between 30 and 39 years, and 16% were above 40 years of age. Over half of the participants (56%) were male. The majority were from the paediatric wards (46%), 36% were from the paediatric and neonatal ICU, 12% were from the anaesthesia department, 2% worked in the emergency department, and another 4% worked in the operation theatres. The majority of the participants were postgraduate residents (36%), 30% were specialists and consultants, and the rest were nursing staff (34%). We found no difference in mean knowledge, attitude, or practice scores between nurses, doctors, and residents or between the genders. The mean scores and standard deviations for knowledge, attitudes, and practice as per designation and gender are shown in Tables 1 and 2.

**Table 1:** Means and standard deviations for Knowledge, attitude, and practice as per designation

Designation		Knowledge Score	Attitude Score	Practice Score
**Nursing staff**	Mean	16.41	6.88	6.71
	Std. Deviation	1.460	.928	1.359
	N	17	17	17
**Doctors**	Mean	16.31	6.69	5.77
	Std. Deviation	2.428	.855	1.536
	N	13	13	13
**Residents**	Mean	15.20	6.40	5.95
	Std. Deviation	2.783	.821	1.538
	N	20	20	20
**Total**	Mean	15.90	6.64	6.16
	Std. Deviation	2.341	.875	1.503
	N	50	50	50

**Table 2:** Means and standard deviations for Knowledge, attitudes, and practice as per gender

Gender		Knowledge_Score	Attitude Score	Practice Score
Female	Mean	15.73	6.68	6.45
	Std. Deviation	2.142	.894	1.262
	N	22	22	22
Male	Mean	16.04	6.61	5.93
	Std. Deviation	2.516	.875	1.654
	N	28	28	28
**Total**	Mean	15.90	6.64	6.16
	Std. Deviation	2.341	.875	1.503
	N	50	50	50

The various departments were categorized into three 1. Paediatric ward and infection control department 2. Intensive care unit 3.Anesthesia/operation theater/Emergency. There was a significant difference in knowledge and Practice scores between the participants of these three departments. There was a significant difference in knowledge and practice scores between the Intensive care unit & the Paediatric ward and infection control department, with the maximum scores in knowledge and practice among participants from the intensive care unit. The mean scores and standard deviations for Knowledge, attitudes, and practice are illustrated in Table 3.

**Table 3:** Means and standard deviations for Knowledge, attitudes, and practice as per departments

Department		Knowledge _Score	Attitude Score	Practice Score
Paediatric ward and infection control department	Mean	15.10	6.48	5.69
	Std. Deviation	2.677	.871	1.417
	N	29	29	29
Intensive care unit	Mean	17.25	6.83	7.42
	Std. Deviation	.622	.835	.515
	N	12	12	12
Anesthesia /operation theater/Emergency	Mean	16.67	6.89	6.00
	Std. Deviation	1.500	.928	1.803
	N	9	9	9
Total	Mean	15.90	6.64	6.16
	Std. Deviation	2.341	.875	1.503
	N	50	50	50

Logistic regression analysis for dependent variables (knowledge and attitude) and independent variables like age, gender, designation, and departments was not significant.

The demographic details of the 40 participants in the qualitative study are as follows. They were aged between 28 and 55 years; 40% had a bachelor’s degree in nursing, 40% had a bachelor’s degree in medicine, 8% had a postgraduate degree in medicine, and 12% had a super specialty degree in paediatric surgery. Equal proportions (40% each) were nurses and postgraduate residents, and 20% worked as consultants in departments like the paediatric medical ward (68%), surgical ward (4%), ICU. (64%), and the infection control department (4%). Most of the participants were from Saudi Arabia (48%) and India (40%); the rest were from the Philippines (10%) and Egypt (10%). The themes, categories, and codes developed in the second phase of the study are presented in Table 4.

**Table 4:** Codebook depicting the themes, categories, and codes (To be placed after the mention of Table 4 in the manuscript text

Theme	Categories	Codes
**1. Knowledge of HAI and infection control**	1.1 Knowledge of HAI	1.1.1 Aware of HAI
		1.1.2 Aware of definition and significance of HAI
	1.2 Types of HAI	1.2.1 Aware of different types of HAI
		1.2.2 Able to identify
	1.3 HAI and infection control	1.3.1 Prevention of HAI
		1.3.2 Importance of infection control
		1.3.3 Association between HAI and infection control
	1.4 Infection control measures in the hospital	1.4.1 Aware of different infection control measures
		1.4.1 Aware of infection control department in the hospital
**2. Infection control measures in practice**	2.1 Hand hygiene	2.1.1 Aware of importance of hand hygiene
		2.1.2 Hand hygiene practised by all HCWs
		2.1.3 5 moments of hand hygiene technique followed
		2.1.4 Hand hygiene week programme
	2.2 Catheter care	2.2.1 Aware of catheter-associated infections-vascular/urinary catheters
		2.2.2 Aware of aseptic catheter insertions
		2.2.3 Care of catheters with care bundles followed
		2.2.4 Checklist followed
		2.2.5 Catheter insertions strictly followed by doctors only
	2.3 PPE	2.3.1 Aware of PPE and its benefitsin infection control
		2.3.2 PPE used only in ICU/isolation room
	2.4 Waste/Sharps disposal	2.4.1 Aware of importance of safe disposal of sharps and waste
		2.4.2 Safe disposal of sharps practised by all HCWs
		2.4.3 Adequate training regarding safe disposal
		2.4.4 Aware of importance of safe disposal of hospital- generated waste
		2.4.5 Proper segregation of waste and colour coding practised
	2.5 Sterilisation and disinfection measures	2.5.1 Aware of the importance of disinfection and sterilisation for infection control
		2.5.2 Proper sterilisation and disinfection procedures followed by all personnel
		2.5.3 Adequate disinfection for blood spills and chemical spills in an ICU setup followed
	2.6 Updates on HAI and infection control	2.6.1 Workshop and seminars conducted once a year for doctors and nurses
		2.6.2 Orientation programme on infection control for residents at the beginning of residency
		2.6.3 Weekly education programme for nurses on HAI and infection control
**3. Gaps in infection control measures and HAI**	3.1 Knowledge	3.1.1 Lack of education about HAI at the start of the residency
		3.1.2 Lack of knowledge about the different types of HAI
	3.2 Communication	3.2.1 Lack of communication between the administrator and hospital personnel on conducting adequate workshops and seminars on infection control and HAI
	3.3 Waste disposal	3.3.1 Lack of awareness of the means of disposal of hospital-generated waste
	3.4 PPE	3.4.1 Lack of proper training on PPE
		3.4.2 PPE used only in ICU
4. **Requirements**	4.1 Workshops and seminars	4.1.2 Need for hands-on catheter insertion training for residents
		4.1.3 Regular workshops and hands-on training on PPE and hand hygiene
	4.2 Waste disposal	4.2.1 Create awareness regarding the means of disposal of hospital-generated waste among all HCWs
	4.3 Sterilisation and disinfection	4.3.1 Educate all HCWs about blood spills and HAI
		4.3.2 Include other paramedical staff in training and workshops on HAI and infection control
	4.4 Change in protocols	4.4.1 Train nurses in aseptic insertion of vascular and urinary catheters
		4.4.2 Follow the disinfection protocol for blood/chemical spills in all areas of the hospital

### Theme 1: knowledge of HAIs and infection control measures

Participants had some idea of the definition of HAIs, their significance, and their different types, such as central line catheter-associated infections, catheter-associated urinary tract infections, ventilator-associated pneumonia, and surgical site infections.

*'My understanding is that they are infections transmitted or acquired by a patient as a result of being in the hospital.'* (P1: paediatrics consultant)

### Theme 2: infection control measures in practice

The hospital implemented infection control measures, such as hand hygiene, usage of PPE, sterilisation/disinfection, and waste and sharps disposal, and most HCWs practiced these measures. Nurses had complete knowledge and practice of maintenance bundles for the care of intravascular lines and urinary catheters. The hospital also held annual seminars on HAIs for nurses and physicians.

*'Hand hygiene is very important. I think it is the key to preventing HAIs'.* (P13: paediatrics consultant)

*'Yeah, seminars, almost annually and even if you [already] got a certificate for that.'* (P5: nurse)

Participants knew of the importance of using PPE, such as masks, caps, protective gowns, and eye goggles. However, they felt that using PPE was necessary only in ICUs and isolation rooms, or when performing procedures.

*'We must wear PPE, like gowns, masks, gloves, and a head cap, in isolation rooms.'* (P9: paediatrics resident).

### Theme 3: gaps in infection control measures

Many of the non-Saudi participants could define HAIs and were aware of the different types. However, Saudi participants, especially junior residents, did not know of the types of HAIs.

*'Sorry, I am not aware of the different types of HAI. I don't know,'.* (P8: paediatrics resident)

Participants believed that the use of PPE is restricted to isolation rooms and ICUs. Furthermore, they received no training in the use of PPE.

*'I have not received proper training on PPE.'* (P1: paediatrics consultant)

Many participants were unaware of the process of hospital-generated waste management or the management of blood and chemical spills. Nurses tended to have a better idea than doctors and junior residents, as they often deal with blood spills using a blood spill kit.

*'Yes, we have a spill kit, especially for biological [spills] …* '. (P2: nurse)

One paediatrics resident could only guess as to the procedure to be followed in case of a chemical/blood spill.

*'I think they wipe it.*' (P8: paediatrics resident)

### Theme 4: required implementation

The participants felt that all HCWs should take part in regular workshops/seminars every three months and hands-on training in hand hygiene techniques, use of PPE, and blood spill management.

*'I think there should be workshops and awareness programmes from time to time, perhaps every three months.'* (P19: Paediatric consultant)

Many participants indicated that paramedical staff should be educated on HAIs and infection control, as they also play a significant role in the process.

### VRE

The third phase of the study comprised of medical procedure VRE sessions performed in the intensive care unit by teams of nurses and doctors (Video sample of the procedure in Additional file 3). Analysis of the video recordings of these procedures by the focus group yielded a single theme: gaps in infection control measures. Table 5 illustrates the codebook featuring the theme, categories, and codes.

**Table 5:** Codebook depicting the theme, categories, and codes of VRE

Theme	Categories	Codes
1. **Gaps in infection control measures**	1.1. A lapse in handwashing technique	1.1.1. Time allotted for handwashing was less than 30 s before and after a sterile procedure
		1.1.2. 5 moments of hand hygiene technique not followed
	1.2. Lack of proper usage of PPE	1.2.1 Surgical masks did not cover the entire beard area of the male resident during the procedure
		1.2.2. The female HCW wore a face cover instead of a surgical mask during a sterile procedure
	1.3. Crossover of non-sterile items into the sterile zone	1.3.1 No zone of demarcation between sterile and non-sterile areas
		1.3.2. Contamination of sterile instruments with non-sterile instruments

PPE: Personal protective equipment; HCW: healthcare worker

### Lapses in handwashing technique

Most of the residents performed hand washing for less than 30 s. They, however, did not follow the five moments of hand hygiene, as advocated by the World Health Organization (WHO) before commencing a sterile procedure. However, the nurses doing the procedures in comparison demonstrated an impeccable handwashing technique before starting the sterile procedure.

### Lack of proper usage of PPE

One of the resident’s mask failed to cover his beard entirely and was not tied securely. In the procedure performed by the nursing personnel, neither the nursing assistant nor the primary nurse (who was performing the procedure) wore a mask; instead, both wore only the face-covering veil (the religious custom among women in Saudi Arabia). The infection control nurse in the focus group agreed that this habit was widespread, irrespective of the repercussions.

### Crossover of non-sterile items into the sterile zone

During the sterile procedures, numerous instances of crossover of non-sterile instruments into sterile areas were observed. For example, in one procedure, normal saline was taken from an open, unsterile saline bottle. There was no line of demarcation between the sterile and non-sterile zone.

### Feedback session

In the feedback session with the participants, the residents acknowledged their lapse in the handwashing technique. They felt that they had to do more to implement what was taught by the infection control department.

**Resident:***'Actually, there are reminders for the steps of hand hygiene … . But I have to practise myself'*.

One particular resident also mentioned that he was pressed for time as he was performing the procedure on a paediatric patient, and thus was in a hurry to finish before the anaesthesia wore off.

During the feedback sessions with the nurses, one of the nurses felt that her face cover was good enough and could double as a surgical mask.

**Nurse**: *'I am wearing a face cover, which is as good as a surgical mask.'*

When probed further, she also mentioned that it is cumbersome to remove her face cover and then wear the mask. The participants also acknowledged the problem of mixing sterile and non-sterile items and said that they would not repeat it in the future. The solutions suggested by the focus group following the reflective sessions are elaborated in Table 6.

**Table 6:** Problems and solutions identified through the VRE methodology

Problems	Solutions	
A lapse in handwashing technique	Educate HCW through videos demonstrating correct and incorrect techniques and infection control practices	Issuance of memos and warnings from the higher authority to the HCWs who do not follow the infection control protocols
Lack of proper usage of PPE.	Make it mandatory for all nurses to wear a surgical mask over the face cover (if worn) during procedures	
Crossover of non-sterile items into the sterile zone	Have an observer from the infection control unit oversee the sterile procedures performed during intensive care unit setup	

H.C.W.: healthcare worker; PPE: personal protective equipment

## Discussion

The quantitative data revealed no significant differences in the knowledge, attitudes, and practices related to infection control among nurses, residents, and senior practitioners and between the gender. However, the participants from the intensive care units had a better score in the knowledge of HAI and the practice of infection control measures. Our finding is contrary to results from other studies that demonstrated poor knowledge of HAI and non-compliance in infection control measures among intensive care unit HCWs [29, 30].

Another KAP study on hand hygiene among HCWs from Al Qassim, Saudi Arabia, reported excellent knowledge but rather poor compliance with the practice of infection control measures and standard precautions [31].

The U.S. Centers for Disease Control and Prevention (CDC) emphasizes the need for HCWs to have sound Knowledge of HAIs and their different types to ensure adequate prevention and control [32]. The qualitative data also made it clear that participants had a sound understanding of the definition of HAIs, the various types, and which types are most common in their hospital, as well as the overall importance of adequate infection control measures.

However, several junior residents in the study demonstrated a lack of knowledge of the different types of HAIs. One recent study revealed the insufficiency of infection control training in medical schools [33]. Another KAP survey from Riyadh on hand hygiene done on three groups of students (medical/nursing/respiratory therapy students) concluded that knowledge of hand hygiene was excellent among all the groups with highest among the nursing and lowest among respiratory therapy students. It was also concluded that good knowledge was associated with good compliance with hand hygiene [34]. A KAP survey from neighboring Namibia reported better KAP scores for medical students as compared to the nursing students. The study emphasized the need to impart knowledge on infection and infection control measures earlier in the curriculum of the students, as we have done in our study [35].

Medical and nursing students in Saudi Arabia are introduced to HAIs and infection control through short lectures in the third year of medical school and the first year of nursing school, respectively. However, they have little access to hospitals during this phase of their studies. We suggest that a practical orientation on infection control measures, along with lectures in teaching hospitals, would benefit students. The training might include weekly practical demonstrations (at least one hour), practice sessions (one hour), presentations (one hour), and a journal club (one hour) for four weeks in the third year of medical school and the first year of nursing school, as a complement to regular lectures. These sessions should focus on the demonstration of hand hygiene techniques, correct usage of PPE, sterilization/disinfection measures, and waste and sharps disposal measures.

Furthermore, short multiple-choice question exams or objective structured practical examinations at the end of these training sessions could also be useful assessments. Intervention at this point is necessary because, during their clinical rotations, students are more focused on clinical cases than on learning infection control measures. Training students at this early stage may help produce medical personnel who are knowledgeable in HAIs and infection control measures.

Well-devised infection control programmes are necessary for preventing HAIs [36]. In our study, participants were aware of the existence of an infection control department in their hospital that functioned in line with CDC guidelines. Studies have demonstrated that many HCWs have inadequate motivation for practicing handwashing techniques and that there is a lack of regular monitoring of compliance with handwashing practices. The lack of motivation may arise from a lack of positive feedback for proper handwashing practices [37]. In our study, regular monitoring of handwashing compliance was conducted, and most HCWs stated that they performed adequate handwashing procedures; this was especially true of nurses, who also actively monitored handwashing practices of other HCWs. The motivation was further boosted via an annual ‘hand hygiene week,’ where the most active participants were rewarded. However, the VRE results demonstrated an apparent lapse in handwashing techniques among residents as compared to nurses. This behaviour has also been reported by many international studies, where compliance with handwashing techniques and recommendations were lower among doctors as compared to other HCWs [38].

The CDC has stressed the need for the availability of different combinations of PPE in ICUs, isolation wards, and other areas in the hospital [39]. All the participants were aware of the importance of PPE but felt usage was only necessary for isolation wards or the ICU. We believe that these problems can be dealt with via education and training regarding the proper use of PPE, as part of an update on infection control practices. The participants also claimed that although gloves and masks were available, there was usually a shortage of gowns, as reported by similar studies where the nonavailability of PPE is a concern [40–42]. The lack of availability of PPE has been brought to the notice of the infection control unit. They, in turn, have alerted the hospital authorities who have promised to address these lacunae.

According to the National Institute for Occupational Safety and Health, workplace needlestick injuries can occur through improper sharps disposal [43]. In our study, nurses were aware of and practiced safe disposal of sharps. Nurses also tended to make sure it was practiced by all HCWs, including doctors, which helped in achieving overall compliance.

HCWs should be mindful of the management of waste, which includes generation, segregation, storage, transport, and disposal [44]. While all participants were aware of the separation of hospital-generated waste and the system of color-coding, very few were aware of the waste management process. We believe that the infection control department should develop training programmes on waste management. Action in this regard should be swift, as ineffective hospital waste management can lead to outbreaks of resistant organisms in the community and, thereby, a serious public health issue. A cross-sectional survey conducted in Saudi Arabia also concludes the fact that extensive training programmes should be in place for the HCWs, especially sanitary workers in hospital settings [45].

Relatedly, hospital policy should ensure that general cleaning, sterilization, and disinfection techniques are strictly followed [46]. HCWs should be aware of how to clean blood spills and blood-contaminated fluids with gloves and the use of internationally approved disinfectants [47]. In the present study, both doctors and nurses were aware of the importance of sterilization and disinfection measures. Nurses were also aware of how to manage chemical/blood spills and followed strict protocols for disinfection. However, several of the junior residents were ignorant of the procedures for chemical/blood spill management as they believed it was not their responsibility. Junior residents should take on the responsibility of educating themselves in the management of blood spills. We, as authors, believe that HCWs, in general, should be trained in blood spill management through seminars and workshops. In our study, as part of the orientation programme, seminars, and presentations on HAIs and infection control were conducted annually by the hospital’s infection control board. However, many of the participants felt that workshops and hands-on training programmes every three months would help update their knowledge. We, the authors believe that intensive training sessions, seminars, journal clubs, and competency assessment tests for all HCWs, including paramedical staff, every three months would ensure that they are equipped with the latest information on HAIs and infection control.

The quantitative and qualitative analysis demonstrated excellent knowledge, practices, and attitudes of HCWs towards HAI and infection control. However, the VRE noted that several residents and nurses did not have complete working knowledge of HAIs nor used the PPE appropriately.

Following the feedback and reflective session, a participant (nurse) acknowledged her lapse in protocol and said that she would wear a surgical mask over her face cover in the future. Similarly, the residents were contrite and promised to follow better infection control measures. The focus group strongly emphasized to the nurse that the face-covering veil is a part of the daily dress code and not a piece of PPE like a surgical mask or surgical gown. As per the guidelines of the Ministry of Health, the surgical mask or N95 respirator should be worn behind the veil or face cover, and then a face shield can be worn over the veil [14, 48]. However, it is evident in our study that nurses and female residents in MERS-CoV endemic areas do not follow these guidelines thoroughly.

Published studies have affirmed that hand hygiene and PPE are essential aspects of infection control in the containment of MERS-CoV outbreaks [49, 50]. In our research, PPE, which plays a significant role in the control of airborne diseases, was not being used appropriately, nor were the five moments of hand hygiene implemented adequately, as demonstrated by the VRE findings. This finding is contrary to the qualitative results, which claimed that sterilization and disinfection measures were followed impeccably.

The crossover of unsterile materials into sterile areas was another problem identified in the video sessions. A similar study conducted in Australia using VRE also reported this problem. It concluded that HCWs should better understand boundary and buffer zones (clean areas) to prevent contamination and the unnecessary crossover of non-sterile items into the sterile zone. They should also prepare well ahead of the procedure to ensure smooth movement across boundaries and fair usage of PPE, and practice effective hand hygiene [51]. Following the reflective sessions, the participants acknowledged their lapses concerning crossover and were open to following strict protocols and changing their working methods.

The solutions to the problems suggested by the focus group, and the details of the reflective sessions in our study, will be presented to the infection control unit and the respective administrative departments to allow for further scrutiny of infection control protocols and practices currently in place. One recent study noted that video-assisted monitoring of clinicians and HCWs could ensure that the HCWs can visualize their flaws and better appreciate current infection control protocols. Regarding the present study, the VRE results can enable the hospital’s infection control unit and administrative board to reflect on the limitations of infection control protocols and the feasibility of implementing more effective infection control practices [52]. An effective way to overcome the lapses is through constant monitoring of HCWs through video footage of sterile procedures.

A cause for concern is the recent reporting of MERS-CoV in parts of Saudi Arabia (Abha and Riyadh) and the new COVID-19 outbreak [53]. As already mentioned, one of the ways to contain the infection is through the effective use of PPE. As per a study conducted in Abha, Saudi Arabia, the Physicians felt that surgical masks were more effective in the prevention of the spread of MERS-CoV as compared to other forms of PPE [50]. Our study implies a lapse in the use of PPE, especially the surgical face masks. It can be argued that if this lapse is not handled appropriately, it can help in the fast spread of airborne diseases like MERS-CoV and COVID-19 in the region as well as the country. Similar reports in the failure of the usage of PPE from different centers can make our argument and findings stronger to gain the attention of the concerned medical authorities and imply strict repercussions to curb this substandard practice.

## Conclusion

To our knowledge, this is the first mixed-methods study on HAI and infection control measures in Saudi Arabia. The sparse knowledge of HAIs, their types, and disinfection measures among junior residents and the process of waste management among other HCWs, failure to engage in boundary maintenance between sterile and non-sterile areas, the inability to observe proper hand hygiene measures, and the careless use of face covers instead of surgical masks were some of the significant findings. The solutions provided by the focus group will assist the infection control unit and administration of this tertiary hospital in bringing about a change in the current policies and infection control strategies. However, a significant limitation of our study is that the findings apply only to this one facility. There is a need to conduct future multicentre studies to replicate our results, which will help bring about changes in current practices, especially improvement in HCWs’ Knowledge through intensive educational programmes.

## Supplementary information

**Additional file 1.** Questionnaire for quantitative data. A detailed questionnaire consisting of 19 knowledge questions, ten practice-related questions, and 12 attitude questions

**Additional file 2.** Interview guide to collect qualitative data. Detailed guide with questions on different types of healthcare-associated infections and infection control practices.

**Additional file 3.** Video recording of a procedure. Video recording of a procedure done by the resident and the assistant nurse.

**Additional file 4.** Statistical analysis. Detailed statistical analysis

## Data Availability

The datasets used in the current study are available from the corresponding author on a reasonable request.

## Abbreviations

HAIs: Healthcare-associated infections
HCWs: Healthcare workers
VRE: Video-reflexive ethnography
PPE: Personal protective equipment
MERS-CoV: Middle East Respiratory Syndrome coronavirus
GCC: The Gulf Cooperation Council
ICU: Intensive care unit
P1: Paediatric consultant
P13: Paediatric consultant
P9: Paediatric resident
P5: Nurse
P8: Paediatric resident
P2: Nurse
P19: Paediatric consultant
WHO: World Health Organization
CDC: Centers for Disease Control and Prevention
EP: Esther Paul
CB: Caryl Beynon
IA: Ibrahim Alzaydani
HC: Harish Chandramoorthy
AH: Ahmed Hakami
AA: Ali Assiri
AM: Abdullah Misfer
SA: Sarah Alsheri
