# Supplementary material for: Healthcare workers’ perspectives on healthcare-associated infections and infection control practices: a video-reflexive ethnography study in the Asir region of Saudi Arabia
Source: Antimicrob Resist Infect Control. 2020 Jul 16;9:110. doi: 10.1186/s13756-020-00756-z (PMC7363991; doi:10.1186/s13756-020-00756-z)
Supplement: Supplementary file 1 — Additional file 1. Questionnaire for quantitative data. A detailed questionnaire consisting of 19 knowledge questions, ten practice-related questions, and 12 attitude questions [file 13756_2020_756_MOESM1_ESM.docx]

**Additional file 1**

**Questionnaire for quantitative data**

**Title:** Awareness of Hospital acquired infections among health-care personnel and infection control measures practiced.

| Name: | Age: | Gender: |
| --- | --- | --- |
| Department: | Speciality: | Designation: |

1.Do you Know the definition of Hospital-acquired infections: Y/N

2.List down the types of Hospital-acquired infection

1. 2. 3. 4. 5.

3.Do you think there is an association between hand hygiene and Hospital-acquiredinfections Y/N

4. Are you aware of the five movements of hand washing technique? Y/N

5. Are you aware of the importance of the safe disposal of sharps? Y/N

6. Is this being followed in your workplace? Y/N

7. Are Sharp injuries being reported? Y/N

8.Are you aware of the importance of safe disposal of hospital generated waste? Y/N

9. Is color coding segregation of waste being followed in your Clinics? Y/N

10. Do you think sterilization and disinfection measures are followed in your Hospital? Y/N

11. Can this reduce hospital-acquired infections? Y/N

12. Are you aware of the procedures followed to manage blood spills and bodily fluid spill? Y/N

13.Is this being followed in your workplace? Y/N

14. Are you aware of the different chemical disinfectant in use? Y/N

15. When do you think sodium hypochlorite solution is required to contain a blood spill?

16. Are you aware that there are different procedures followed as per the size of the spill (Small<10cms, Large> 10cms )? Y/N

17. Is hand hygiene followed following the decontamination? Y/N

18.Do you think this is important? Y/N

19.Are you aware of the procedure followed in the disposal of the soiled materials used to decontaminate the blood spill/body fluid spill areas? Y/N

**ATTITUDE**

1.How many workshops and seminars you have attended on HAI in the past twoyears:

2.Do you think there is a need for education of healthcare workers on HAI and infection control measures? Y/N

3.Who is in charge of managing blood spill and body fluid spill? (Tick in appropriate boxes

Consultants/specialist Residents Nurses Interns Health worker

4. Do you think you need to use Personal protective equipment for blood spill/body fluid spill management? Y/N

5. Do you think hands-on training and workshop programs focused on infection control would be helpful to all the health personnel at your workplace? Y/N

6.What should be the frequency of these educational programs/ workshops and seminars on HAI Everythree months Every six months Annually

7. Do you think all the health care workers follow all the five steps of hand washing technique?

8. How can you educate your fellow workers / Nursing staff and students (Tick in appropriate boxes)

You set an example: Seminars Lectures Workshops

Orientation programs Follow hand hygiene week

9. Do you think the direct observation of the Health personnel will help in improving the practice of Hand hygiene?

10. Do you think using personal protective equipment like face masks, gloves and gowns are important in your practice Y/N

**PRACTICE**

1 How many times during the day do you practice Hand hygiene?

2. What do you use to decontaminate your hands? Soap Alcohol rub

3. Are wash basin and soap available at appropriate places in your clinics? Y/N

4. Are alcohol rubs available for use at your workplace and clinics? Y/N

5. Do you follow the five steps when you wash your hands? Y/N

6. How long do you wash your hands with soap and water?

7. How long do you take to decontaminate your hands with alcohol rub?

8. Have you been given Hepatitis B vaccine Y/N
