# Supplementary material for: Healthcare workers’ perspectives on healthcare-associated infections and infection control practices: a video-reflexive ethnography study in the Asir region of Saudi Arabia
Source: Antimicrob Resist Infect Control. 2020 Jul 16;9:110. doi: 10.1186/s13756-020-00756-z (PMC7363991; doi:10.1186/s13756-020-00756-z)
Supplement: Supplementary file 2 — Additional file 2. Interview guide to collect qualitative data. Detailed guide with questions on different types of healthcare-associated infections and infection control practices. [file 13756_2020_756_MOESM2_ESM.docx]

**Additional file 2**

**Interview guide**

**Research question**: What are the healthcare workers ‘perspectiveson Health Care-associated infections and infection control measures with a focus on hand hygiene

**Age**

**Gender:**

**Nationality:**

**Qualification and position**

Let’s first talk a bit about you. Which department you work in/ what is your specialisation?

What do you understand by the term health-care-associated infections (HAI)? and the infection control measures

Have you attended any seminars and workshops on HAI?

When did you first learn about HAI at what level of your medical studies? Or nursing studies?

What is your opinion about the need for education of healthcare workers on HAI and infection control measures?

Are you aware of the different HAI? Can you describe them in detail?

What do you understand by Hand decontamination?

Are you aware of the association between hand hygiene and HAI?

What is your take on hand hygiene playing a major role in reducing the transmission of multi-drug resistant organisms?

Are you aware of the WHO recommended " My five moments for hand hygiene model? Can you describe the steps involved?

What sort of hand hygiene is available in hospital/ Department?

Which in your opinion is better soap and water or Alcohol-based rubs?

How long do you think one needs to wash his hands with soap and water?

What about alcohol rubs? How long do you think you need to clean your hands with the alcohol-basedrubs?

What sort of and hygiene product do the other health personnel and students use?

What is your knowledge of personal protective equipment? Gloves, face masks etc Can you describe any awareness programs conducted in the hospital of your clinical rotation?

Can you describe the procedure for the safe disposal of sharps? Are you aware that all the healthcare workers follow the procedure in your hospital?

What is your understanding of the safe disposal of the hospital generated waste? Will this help in infection control?

Are you aware of the segregation methods followed in the hospital?

Are you aware of the methods used to dispose of the hospital generated waste?

What is the procedure followed here?

Can you describe the recommended procedures for sterilization and disinfection measures followed in your hospital?

Describe how this can help to contain and control HAI.

Are you aware of the procedures followed to manage blood spills and bodily fluid spill?

Are you aware of the different chemical disinfectant in use?

When do you think sodium hypochlorite solution is required to contain a blood spill?

Is hand hygiene followed following the decontamination? Do you think this is important?

Do you think hands-on training and workshop programs focused on hand hygiene and blood spill management would be helpful to all the health personnel at your workplace?

Do you think enough education is given about HAI during your study period?

Do you think education about HAI and infection control should be stressed upon during the orientation before the start of your work period ?
