# Supplementary material for: Healthcare workers’ perspectives on healthcare-associated infections and infection control practices: a video-reflexive ethnography study in the Asir region of Saudi Arabia
Source: Antimicrob Resist Infect Control. 2020 Jul 16;9:110. doi: 10.1186/s13756-020-00756-z (PMC7363991; doi:10.1186/s13756-020-00756-z)
Supplement: Supplementary file 4 — Additional file 4. Statistical analysis. Detailed statistical analysis [file 13756_2020_756_MOESM4_ESM.docx]

**Statistical calculations for KAP**

| **Report** | | | | |
| --- | --- | --- | --- | --- |
| Designation_Code | | Know_Score | Atti_Score | Pract_Score |
| Nursing staff | Mean | 16.41 | 6.88 | 6.71 |
|  | Std. Deviation | 1.460 | .928 | 1.359 |
|  | N | 17 | 17 | 17 |
| Doctors | Mean | 16.31 | 6.69 | 5.77 |
|  | Std. Deviation | 2.428 | .855 | 1.536 |
|  | N | 13 | 13 | 13 |
| Residents | Mean | 15.20 | 6.40 | 5.95 |
|  | Std. Deviation | 2.783 | .821 | 1.538 |
|  | N | 20 | 20 | 20 |
| Total | Mean | 15.90 | 6.64 | 6.16 |
|  | Std. Deviation | 2.341 | .875 | 1.503 |
|  | N | 50 | 50 | 50 |

**Kruskal-Wallis Test**

Since the data doesn't satisfies normality condition ANOVA was not used to test mean difference in various scores between Nurse, Doctors and residents.

| **Ranks** | | | |
| --- | --- | --- | --- |
|  | Designation_Code | N | Mean Rank |
| Know_Score | Nursing staff | 17 | 15.18 |
|  | Doctors | 13 | 15.92 |
|  | Total | 30 |  |
| Atti_Score | Nursing staff | 17 | 16.21 |
|  | Doctors | 13 | 14.58 |
|  | Total | 30 |  |
| Pract_Score | Nursing staff | 17 | 17.88 |
|  | Doctors | 13 | 12.38 |
|  | Total | 30 |  |

| **Test Statistics^a,b^** | | | |
| --- | --- | --- | --- |
|  | Know_Score | Atti_Score | Pract_Score |
| Chi-Square | .056 | .300 | 3.223 |
| df | 1 | 1 | 1 |
| Asymp. Sig. | .814 | .584 | .073 |
| a. Kruskal Wallis Test | | | |
| b. Grouping Variable: Designation_Code | | | |

There is no difference in mean knowledge between Nurse, Doctors and Residents.

There is no difference in mean Attitude between Nurse, Doctors and Residents.

There is no difference in mean practicge between Nurse, Doctors and Residents.

**Below are frequency and cross table of all variables**

**Frequency Table**

| **Age:** | | | | | |
| --- | --- | --- | --- | --- | --- |
|  | | Frequency | Percent | Valid Percent | Cumulative Percent |
| Valid | 24 | 1 | 2.0 | 2.0 | 2.0 |
|  | 25 | 5 | 10.0 | 10.0 | 12.0 |
|  | 26 | 7 | 14.0 | 14.0 | 26.0 |
|  | 27 | 3 | 6.0 | 6.0 | 32.0 |
|  | 28 | 4 | 8.0 | 8.0 | 40.0 |
|  | 29 | 4 | 8.0 | 8.0 | 48.0 |
|  | 30 | 7 | 14.0 | 14.0 | 62.0 |
|  | 31 | 1 | 2.0 | 2.0 | 64.0 |
|  | 32 | 2 | 4.0 | 4.0 | 68.0 |
|  | 33 | 1 | 2.0 | 2.0 | 70.0 |
|  | 34 | 1 | 2.0 | 2.0 | 72.0 |
|  | 35 | 1 | 2.0 | 2.0 | 74.0 |
|  | 37 | 3 | 6.0 | 6.0 | 80.0 |
|  | 38 | 2 | 4.0 | 4.0 | 84.0 |
|  | 40 | 1 | 2.0 | 2.0 | 86.0 |
|  | 43 | 1 | 2.0 | 2.0 | 88.0 |
|  | 45 | 2 | 4.0 | 4.0 | 92.0 |
|  | 47 | 1 | 2.0 | 2.0 | 94.0 |
|  | 49 | 1 | 2.0 | 2.0 | 96.0 |
|  | 50 | 1 | 2.0 | 2.0 | 98.0 |
|  | 55 | 1 | 2.0 | 2.0 | 100.0 |
|  | Total | 50 | 100.0 | 100.0 |  |

| **Gender** | | | | | |
| --- | --- | --- | --- | --- | --- |
|  | | Frequency | Percent | Valid Percent | Cumulative Percent |
| Valid | Female | 22 | 44.0 | 44.0 | 44.0 |
|  | Male | 28 | 56.0 | 56.0 | 100.0 |
|  | Total | 50 | 100.0 | 100.0 |  |

| **Department** | | | | | |
| --- | --- | --- | --- | --- | --- |
|  | | Frequency | Percent | Valid Percent | Cumulative Percent |
| Valid | Anesthesia | 6 | 12.0 | 12.0 | 12.0 |
|  | ER | 1 | 2.0 | 2.0 | 14.0 |
|  | IPC | 6 | 12.0 | 12.0 | 26.0 |
|  | NICU | 8 | 16.0 | 16.0 | 42.0 |
|  | NPCU | 1 | 2.0 | 2.0 | 44.0 |
|  | OR | 2 | 4.0 | 4.0 | 48.0 |
|  | Paediatric | 4 | 8.0 | 8.0 | 56.0 |
|  | PICU | 3 | 6.0 | 6.0 | 62.0 |
|  | PMW | 19 | 38.0 | 38.0 | 100.0 |
|  | Total | 50 | 100.0 | 100.0 |  |

| **Speciality** | | | | | |
| --- | --- | --- | --- | --- | --- |
|  | | Frequency | Percent | Valid Percent | Cumulative Percent |
| Valid | Anesthesia | 6 | 12.0 | 12.0 | 12.0 |
|  | Cardiology | 2 | 4.0 | 4.0 | 16.0 |
|  | ER | 1 | 2.0 | 2.0 | 18.0 |
|  | Infectious diseases | 1 | 2.0 | 2.0 | 20.0 |
|  | Nephrology | 1 | 2.0 | 2.0 | 22.0 |
|  | NICU | 3 | 6.0 | 6.0 | 28.0 |
|  | Nurse | 10 | 20.0 | 20.0 | 48.0 |
|  | Paediatric | 22 | 44.0 | 44.0 | 92.0 |
|  | Pathologist | 1 | 2.0 | 2.0 | 94.0 |
|  | PICU | 3 | 6.0 | 6.0 | 100.0 |
|  | Total | 50 | 100.0 | 100.0 |  |

| **Designation** | | | | | |
| --- | --- | --- | --- | --- | --- |
|  | | Frequency | Percent | Valid Percent | Cumulative Percent |
| Valid | Anaesthetist | 2 | 4.0 | 4.0 | 4.0 |
|  | Cardiologist | 2 | 4.0 | 4.0 | 8.0 |
|  | Consultant | 4 | 8.0 | 8.0 | 16.0 |
|  | GP | 1 | 2.0 | 2.0 | 18.0 |
|  | IPC Nurse practitioner | 3 | 6.0 | 6.0 | 24.0 |
|  | Nurse | 13 | 26.0 | 26.0 | 50.0 |
|  | OR reception | 1 | 2.0 | 2.0 | 52.0 |
|  | Resident | 19 | 38.0 | 38.0 | 90.0 |
|  | Senior registar | 1 | 2.0 | 2.0 | 92.0 |
|  | Specialist | 4 | 8.0 | 8.0 | 100.0 |
|  | Total | 50 | 100.0 | 100.0 |  |

Knowledge

| **Do you Know the definition of Hospital-acquired infections:** | | | | | |
| --- | --- | --- | --- | --- | --- |
|  | | Frequency | Percent | Valid Percent | Cumulative Percent |
| Valid | Wrong answer | 3 | 6.0 | 6.0 | 6.0 |
|  | Correct answer | 47 | 94.0 | 94.0 | 100.0 |
|  | Total | 50 | 100.0 | 100.0 |  |

| **List down the types of Hospital-acquired infection** | | | | | |
| --- | --- | --- | --- | --- | --- |
|  | | Frequency | Percent | Valid Percent | Cumulative Percent |
| Valid | Wrong answer | 28 | 56.0 | 56.0 | 56.0 |
|  | Correct answer | 22 | 44.0 | 44.0 | 100.0 |
|  | Total | 50 | 100.0 | 100.0 |  |

| **Do you think there is an association between hand hygiene and Hospital-acquired infections** | | | | | |
| --- | --- | --- | --- | --- | --- |
|  | | Frequency | Percent | Valid Percent | Cumulative Percent |
| Valid | Wrong answer | 3 | 6.0 | 6.0 | 6.0 |
|  | Correct answer | 47 | 94.0 | 94.0 | 100.0 |
|  | Total | 50 | 100.0 | 100.0 |  |

| **Are you aware of the five movements of hand washing technique?** | | | | | |
| --- | --- | --- | --- | --- | --- |
|  | | Frequency | Percent | Valid Percent | Cumulative Percent |
| Valid | Correct answer | 50 | 100.0 | 100.0 | 100.0 |

| **Are you aware of the importance of the safe disposal of sharps?** | | | | | |
| --- | --- | --- | --- | --- | --- |
|  | | Frequency | Percent | Valid Percent | Cumulative Percent |
| Valid | Correct answer | 50 | 100.0 | 100.0 | 100.0 |

| **Is safe disposal of sharps being followed in your workplace?** | | | | | |
| --- | --- | --- | --- | --- | --- |
|  | | Frequency | Percent | Valid Percent | Cumulative Percent |
| Valid | Wrong answer | 1 | 2.0 | 2.0 | 2.0 |
|  | Correct answer | 49 | 98.0 | 98.0 | 100.0 |
|  | Total | 50 | 100.0 | 100.0 |  |

| **Are sharp injuries being reported?** | | | | | |
| --- | --- | --- | --- | --- | --- |
|  | | Frequency | Percent | Valid Percent | Cumulative Percent |
| Valid | Wrong answer | 12 | 24.0 | 24.0 | 24.0 |
|  | Correct answer | 38 | 76.0 | 76.0 | 100.0 |
|  | Total | 50 | 100.0 | 100.0 |  |

| **Are you aware of the importance of safe disposal of hospital generated waste?** | | | | | |
| --- | --- | --- | --- | --- | --- |
|  | | Frequency | Percent | Valid Percent | Cumulative Percent |
| Valid | Wrong answer | 2 | 4.0 | 4.0 | 4.0 |
|  | Correct answer | 48 | 96.0 | 96.0 | 100.0 |
|  | Total | 50 | 100.0 | 100.0 |  |

| **Is color coding segregation of waste being followed in your Clinics?** | | | | | |
| --- | --- | --- | --- | --- | --- |
|  | | Frequency | Percent | Valid Percent | Cumulative Percent |
| Valid | Wrong answer | 8 | 16.0 | 16.0 | 16.0 |
|  | Correct answer | 42 | 84.0 | 84.0 | 100.0 |
|  | Total | 50 | 100.0 | 100.0 |  |

| **Do you think sterilization and disinfection measures followed in your Clinics?** | | | | | |
| --- | --- | --- | --- | --- | --- |
|  | | Frequency | Percent | Valid Percent | Cumulative Percent |
| Valid | Wrong answer | 5 | 10.0 | 10.0 | 10.0 |
|  | Correct answer | 45 | 90.0 | 90.0 | 100.0 |
|  | Total | 50 | 100.0 | 100.0 |  |

| **Can sterilization and disinfection measures reduce hospital-acquired infections?** | | | | | |
| --- | --- | --- | --- | --- | --- |
|  | | Frequency | Percent | Valid Percent | Cumulative Percent |
| Valid | Wrong answer | 1 | 2.0 | 2.0 | 2.0 |
|  | Correct answer | 49 | 98.0 | 98.0 | 100.0 |
|  | Total | 50 | 100.0 | 100.0 |  |

| **Are you aware of the procedures followed to manage blood spills and bodily fluid spill?** | | | | | |
| --- | --- | --- | --- | --- | --- |
|  | | Frequency | Percent | Valid Percent | Cumulative Percent |
| Valid | Wrong answer | 4 | 8.0 | 8.0 | 8.0 |
|  | Correct answer | 46 | 92.0 | 92.0 | 100.0 |
|  | Total | 50 | 100.0 | 100.0 |  |

| **Is the above procedure being followed in your workplace?** | | | | | |
| --- | --- | --- | --- | --- | --- |
|  | | Frequency | Percent | Valid Percent | Cumulative Percent |
| Valid | Wrong answer | 4 | 8.0 | 8.0 | 8.0 |
|  | Correct answer | 46 | 92.0 | 92.0 | 100.0 |
|  | Total | 50 | 100.0 | 100.0 |  |

| **Are you aware of the different chemical disinfectant in use?** | | | | | |
| --- | --- | --- | --- | --- | --- |
|  | | Frequency | Percent | Valid Percent | Cumulative Percent |
| Valid | Wrong answer | 13 | 26.0 | 26.0 | 26.0 |
|  | Correct answer | 37 | 74.0 | 74.0 | 100.0 |
|  | Total | 50 | 100.0 | 100.0 |  |

| **When do you think sodium hypochlorite solution is required to contain a blood spill?** | | | | | |
| --- | --- | --- | --- | --- | --- |
|  | | Frequency | Percent | Valid Percent | Cumulative Percent |
| Valid | Wrong answer | 41 | 82.0 | 82.0 | 82.0 |
|  | Correct answer | 9 | 18.0 | 18.0 | 100.0 |
|  | Total | 50 | 100.0 | 100.0 |  |

| **Are you aware that there are different procedures followed as per the size of the spill (Small&lt;10cms, Large&gt; 10cms )?** | | | | | |
| --- | --- | --- | --- | --- | --- |
|  | | Frequency | Percent | Valid Percent | Cumulative Percent |
| Valid | Wrong answer | 17 | 34.0 | 34.0 | 34.0 |
|  | Correct answer | 33 | 66.0 | 66.0 | 100.0 |
|  | Total | 50 | 100.0 | 100.0 |  |

| **After the decontamination procedure is hand hygiene being followed?** | | | | | |
| --- | --- | --- | --- | --- | --- |
|  | | Frequency | Percent | Valid Percent | Cumulative Percent |
| Valid | Correct answer | 50 | 100.0 | 100.0 | 100.0 |

| **Do you think hand hygiene at this time is important?** | | | | | |
| --- | --- | --- | --- | --- | --- |
|  | | Frequency | Percent | Valid Percent | Cumulative Percent |
| Valid | Wrong answer | 1 | 2.0 | 2.0 | 2.0 |
|  | Correct answer | 49 | 98.0 | 98.0 | 100.0 |
|  | Total | 50 | 100.0 | 100.0 |  |

| **Are you aware of the procedure followed in the disposal of the soiled materials used to decontaminate the blood spill/body fluid spill areas?** | | | | | |
| --- | --- | --- | --- | --- | --- |
|  | | Frequency | Percent | Valid Percent | Cumulative Percent |
| Valid | Wrong answer | 12 | 24.0 | 24.0 | 24.0 |
|  | Correct answer | 38 | 76.0 | 76.0 | 100.0 |
|  | Total | 50 | 100.0 | 100.0 |  |

| **Know_Score** | | | | | |
| --- | --- | --- | --- | --- | --- |
|  | | Frequency | Percent | Valid Percent | Cumulative Percent |
| Valid | 10 | 2 | 4.0 | 4.0 | 4.0 |
|  | 11 | 1 | 2.0 | 2.0 | 6.0 |
|  | 12 | 3 | 6.0 | 6.0 | 12.0 |
|  | 13 | 3 | 6.0 | 6.0 | 18.0 |
|  | 14 | 3 | 6.0 | 6.0 | 24.0 |
|  | 15 | 5 | 10.0 | 10.0 | 34.0 |
|  | 16 | 4 | 8.0 | 8.0 | 42.0 |
|  | 17 | 17 | 34.0 | 34.0 | 76.0 |
|  | 18 | 9 | 18.0 | 18.0 | 94.0 |
|  | 19 | 3 | 6.0 | 6.0 | 100.0 |
|  | Total | 50 | 100.0 | 100.0 |  |

Attitude

| **How many workshops and seminars you have attended on HAI in the past two years :** | | | | | |
| --- | --- | --- | --- | --- | --- |
|  | | Frequency | Percent | Valid Percent | Cumulative Percent |
| Valid | Wrong answer | 41 | 82.0 | 82.0 | 82.0 |
|  | Correct answer | 9 | 18.0 | 18.0 | 100.0 |
|  | Total | 50 | 100.0 | 100.0 |  |

| **Do you think there is a need for education of healthcare workers on HAI and infection control measures?** | | | | | |
| --- | --- | --- | --- | --- | --- |
|  | | Frequency | Percent | Valid Percent | Cumulative Percent |
| Valid | Correct answer | 50 | 100.0 | 100.0 | 100.0 |

| **Do you think all the health care workers follow all the five steps of hand washing technique?** | | | | | |
| --- | --- | --- | --- | --- | --- |
|  | | Frequency | Percent | Valid Percent | Cumulative Percent |
| Valid | Wrong answer | 40 | 80.0 | 80.0 | 80.0 |
|  | Correct answer | 10 | 20.0 | 20.0 | 100.0 |
|  | Total | 50 | 100.0 | 100.0 |  |

| **Do you think the direct observation of health personnel will help in improving the practice of Hand hygiene?** | | | | | |
| --- | --- | --- | --- | --- | --- |
|  | | Frequency | Percent | Valid Percent | Cumulative Percent |
| Valid | Wrong answer | 3 | 6.0 | 6.0 | 6.0 |
|  | Correct answer | 47 | 94.0 | 94.0 | 100.0 |
|  | Total | 50 | 100.0 | 100.0 |  |

| **Do you think using personal protective equipment like face masks, gloves and gowns are important in your practice** | | | | | |
| --- | --- | --- | --- | --- | --- |
|  | | Frequency | Percent | Valid Percent | Cumulative Percent |
| Valid | Wrong answer | 2 | 4.0 | 4.0 | 4.0 |
|  | Correct answer | 48 | 96.0 | 96.0 | 100.0 |
|  | Total | 50 | 100.0 | 100.0 |  |

| **Who is in charge of managing blood spill and body fluid spill?** | | | | | |
| --- | --- | --- | --- | --- | --- |
|  | | Frequency | Percent | Valid Percent | Cumulative Percent |
| Valid | Correct answer | 50 | 100.0 | 100.0 | 100.0 |

| **Do you think you need to use Personal Protective Equipments while blood spill/body fluid spill management?** | | | | | |
| --- | --- | --- | --- | --- | --- |
|  | | Frequency | Percent | Valid Percent | Cumulative Percent |
| Valid | Wrong answer | 1 | 2.0 | 2.0 | 2.0 |
|  | Correct answer | 49 | 98.0 | 98.0 | 100.0 |
|  | Total | 50 | 100.0 | 100.0 |  |

| **What in your opinion should be the frequency of these educational programs/ workshops and seminars on HAI ?** | | | | | |
| --- | --- | --- | --- | --- | --- |
|  | | Frequency | Percent | Valid Percent | Cumulative Percent |
| Valid | Wrong answer | 37 | 74.0 | 74.0 | 74.0 |
|  | Correct answer | 13 | 26.0 | 26.0 | 100.0 |
|  | Total | 50 | 100.0 | 100.0 |  |

| **How can you educate your fellow workers / Nursing staff and students** | | | | | |
| --- | --- | --- | --- | --- | --- |
|  | | Frequency | Percent | Valid Percent | Cumulative Percent |
| Valid | Wrong answer | 44 | 88.0 | 88.0 | 88.0 |
|  | Correct answer | 6 | 12.0 | 12.0 | 100.0 |
|  | Total | 50 | 100.0 | 100.0 |  |

| **Do you think hands-on training and workshop programs focused on hand hygiene and blood spill management would be helpful to all the health personnel at your workplace?** | | | | | |
| --- | --- | --- | --- | --- | --- |
|  | | Frequency | Percent | Valid Percent | Cumulative Percent |
| Valid | Correct answer | 50 | 100.0 | 100.0 | 100.0 |

| **Atti_Score** | | | | | |
| --- | --- | --- | --- | --- | --- |
|  | | Frequency | Percent | Valid Percent | Cumulative Percent |
| Valid | 5 | 2 | 4.0 | 4.0 | 4.0 |
|  | 6 | 25 | 50.0 | 50.0 | 54.0 |
|  | 7 | 12 | 24.0 | 24.0 | 78.0 |
|  | 8 | 11 | 22.0 | 22.0 | 100.0 |
|  | Total | 50 | 100.0 | 100.0 |  |

Practice

| **How many times during the day do you practice Hand hygiene?** | | | | | |
| --- | --- | --- | --- | --- | --- |
|  | | Frequency | Percent | Valid Percent | Cumulative Percent |
| Valid | Wrong answer | 21 | 42.0 | 42.0 | 42.0 |
|  | Correct answer | 29 | 58.0 | 58.0 | 100.0 |
|  | Total | 50 | 100.0 | 100.0 |  |

| **What do you use to decontaminate your hands?** | | | | | |
| --- | --- | --- | --- | --- | --- |
|  | | Frequency | Percent | Valid Percent | Cumulative Percent |
| Valid | Wrong answer | 32 | 64.0 | 64.0 | 64.0 |
|  | Correct answer | 18 | 36.0 | 36.0 | 100.0 |
|  | Total | 50 | 100.0 | 100.0 |  |

| **Are wash basin and soap available at appropriate places in your clinics?** | | | | | |
| --- | --- | --- | --- | --- | --- |
|  | | Frequency | Percent | Valid Percent | Cumulative Percent |
| Valid | Wrong answer | 7 | 14.0 | 14.0 | 14.0 |
|  | Correct answer | 43 | 86.0 | 86.0 | 100.0 |
|  | Total | 50 | 100.0 | 100.0 |  |

| **Are alcohol rubs available for use at your workplace and clinics?** | | | | | |
| --- | --- | --- | --- | --- | --- |
|  | | Frequency | Percent | Valid Percent | Cumulative Percent |
| Valid | Wrong answer | 2 | 4.0 | 4.0 | 4.0 |
|  | Correct answer | 48 | 96.0 | 96.0 | 100.0 |
|  | Total | 50 | 100.0 | 100.0 |  |

| **Do you follow the five steps when you wash your hands?** | | | | | |
| --- | --- | --- | --- | --- | --- |
|  | | Frequency | Percent | Valid Percent | Cumulative Percent |
| Valid | Wrong answer | 6 | 12.0 | 12.0 | 12.0 |
|  | Correct answer | 44 | 88.0 | 88.0 | 100.0 |
|  | Total | 50 | 100.0 | 100.0 |  |

| **How long do you wash your hands with soap and water?** | | | | | |
| --- | --- | --- | --- | --- | --- |
|  | | Frequency | Percent | Valid Percent | Cumulative Percent |
| Valid | Wrong answer | 3 | 6.0 | 6.0 | 6.0 |
|  | Correct answer | 47 | 94.0 | 94.0 | 100.0 |
|  | Total | 50 | 100.0 | 100.0 |  |

| **How long do you take to decontaminate your hands with alcohol rub?** | | | | | |
| --- | --- | --- | --- | --- | --- |
|  | | Frequency | Percent | Valid Percent | Cumulative Percent |
| Valid | Wrong answer | 13 | 26.0 | 26.0 | 26.0 |
|  | Correct answer | 37 | 74.0 | 74.0 | 100.0 |
|  | Total | 50 | 100.0 | 100.0 |  |

| **Have you been given Hep B vaccine** | | | | | |
| --- | --- | --- | --- | --- | --- |
|  | | Frequency | Percent | Valid Percent | Cumulative Percent |
| Valid | Wrong answer | 8 | 16.0 | 16.0 | 16.0 |
|  | Correct answer | 42 | 84.0 | 84.0 | 100.0 |
|  | Total | 50 | 100.0 | 100.0 |  |

| **Pract_Score** | | | | | |
| --- | --- | --- | --- | --- | --- |
|  | | Frequency | Percent | Valid Percent | Cumulative Percent |
| Valid | 3 | 3 | 6.0 | 6.0 | 6.0 |
|  | 4 | 6 | 12.0 | 12.0 | 18.0 |
|  | 5 | 7 | 14.0 | 14.0 | 32.0 |
|  | 6 | 7 | 14.0 | 14.0 | 46.0 |
|  | 7 | 18 | 36.0 | 36.0 | 82.0 |
|  | 8 | 9 | 18.0 | 18.0 | 100.0 |
|  | Total | 50 | 100.0 | 100.0 |  |

| **How many times during the day do you practice Hand hygiene?** | | | | | |
| --- | --- | --- | --- | --- | --- |
|  | | Frequency | Percent | Valid Percent | Cumulative Percent |
| Valid | >3 | 2 | 4.0 | 4.0 | 4.0 |
|  | 10. | 13 | 26.0 | 26.0 | 30.0 |
|  | 15. | 4 | 8.0 | 8.0 | 38.0 |
|  | 2. | 1 | 2.0 | 2.0 | 40.0 |
|  | 20. | 3 | 6.0 | 6.0 | 46.0 |
|  | 3-4 times | 1 | 2.0 | 2.0 | 48.0 |
|  | 3. | 4 | 8.0 | 8.0 | 56.0 |
|  | 4. | 2 | 4.0 | 4.0 | 60.0 |
|  | 5-10 | 1 | 2.0 | 2.0 | 62.0 |
|  | 5. | 8 | 16.0 | 16.0 | 78.0 |
|  | 7. | 1 | 2.0 | 2.0 | 80.0 |
|  | 8. | 3 | 6.0 | 6.0 | 86.0 |
|  | After every patient | 2 | 4.0 | 4.0 | 90.0 |
|  | Depends on patient recieved | 1 | 2.0 | 2.0 | 92.0 |
|  | Many | 4 | 8.0 | 8.0 | 100.0 |
|  | Total | 50 | 100.0 | 100.0 |  |

| **What do you use to decontaminate your hands?** | | | | | |
| --- | --- | --- | --- | --- | --- |
|  | | Frequency | Percent | Valid Percent | Cumulative Percent |
| Valid | Alcohol Rub | 23 | 46.0 | 46.0 | 46.0 |
|  | Soap | 9 | 18.0 | 18.0 | 64.0 |
|  | Soap, Alcohol Rub | 18 | 36.0 | 36.0 | 100.0 |
|  | Total | 50 | 100.0 | 100.0 |  |

| **How long do you wash your hands with soap and water?** | | | | | |
| --- | --- | --- | --- | --- | --- |
|  | | Frequency | Percent | Valid Percent | Cumulative Percent |
| Valid | 1 min | 24 | 48.0 | 48.0 | 48.0 |
|  | 10 sec | 2 | 4.0 | 4.0 | 52.0 |
|  | 2 mins | 5 | 10.0 | 10.0 | 62.0 |
|  | 20-30 secs | 5 | 10.0 | 10.0 | 72.0 |
|  | 25 secs | 1 | 2.0 | 2.0 | 74.0 |
|  | 3 mins | 1 | 2.0 | 2.0 | 76.0 |
|  | 30 secs | 1 | 2.0 | 2.0 | 78.0 |
|  | 30-60 secs | 2 | 4.0 | 4.0 | 82.0 |
|  | 40 secs | 1 | 2.0 | 2.0 | 84.0 |
|  | 40-60 secs | 4 | 8.0 | 8.0 | 92.0 |
|  | 40-80 secs | 1 | 2.0 | 2.0 | 94.0 |
|  | 45sec-1 min | 1 | 2.0 | 2.0 | 96.0 |
|  | 8 mins | 1 | 2.0 | 2.0 | 98.0 |
|  | less than 20 secs | 1 | 2.0 | 2.0 | 100.0 |
|  | Total | 50 | 100.0 | 100.0 |  |

| **How long do you take to decontaminate your hands with alcohol rub?** | | | | | |
| --- | --- | --- | --- | --- | --- |
|  | | Frequency | Percent | Valid Percent | Cumulative Percent |
| Valid | 1 min | 7 | 14.0 | 14.0 | 14.0 |
|  | 10 sec | 1 | 2.0 | 2.0 | 16.0 |
|  | 10-20 secs | 1 | 2.0 | 2.0 | 18.0 |
|  | 15 secs | 1 | 2.0 | 2.0 | 20.0 |
|  | 15-20 secs | 1 | 2.0 | 2.0 | 22.0 |
|  | 20 secs | 1 | 2.0 | 2.0 | 24.0 |
|  | 20-30 secs | 11 | 22.0 | 22.0 | 46.0 |
|  | 3 mins | 1 | 2.0 | 2.0 | 48.0 |
|  | 30 secs | 15 | 30.0 | 30.0 | 78.0 |
|  | 40 secs | 1 | 2.0 | 2.0 | 80.0 |
|  | 5 mins | 1 | 2.0 | 2.0 | 82.0 |
|  | 5 secs | 8 | 16.0 | 16.0 | 98.0 |
|  | 5-10secs | 1 | 2.0 | 2.0 | 100.0 |
|  | Total | 50 | 100.0 | 100.0 |  |

| **Age_Grp** | | | | | |
| --- | --- | --- | --- | --- | --- |
|  | | Frequency | Percent | Valid Percent | Cumulative Percent |
| Valid | 20 - 29 yrs | 24 | 48.0 | 48.0 | 48.0 |
|  | 30 - 39 yrs | 18 | 36.0 | 36.0 | 84.0 |
|  | >= 40 yrs | 8 | 16.0 | 16.0 | 100.0 |
|  | Total | 50 | 100.0 | 100.0 |  |

| **Know_Code** | | | | | |
| --- | --- | --- | --- | --- | --- |
|  | | Frequency | Percent | Valid Percent | Cumulative Percent |
| Valid | Adequate Knowledge (50 - 75%) | 12 | 24.0 | 24.0 | 24.0 |
|  | Good knowledge (>75%) | 38 | 76.0 | 76.0 | 100.0 |
|  | Total | 50 | 100.0 | 100.0 |  |

| **Atti_Code** | | | | | |
| --- | --- | --- | --- | --- | --- |
|  | | Frequency | Percent | Valid Percent | Cumulative Percent |
| Valid | Adequate Attitude (50 - 75%) | 39 | 78.0 | 78.0 | 78.0 |
|  | Good Attitude (>75%) | 11 | 22.0 | 22.0 | 100.0 |
|  | Total | 50 | 100.0 | 100.0 |  |

| **Pract_Code** | | | | | |
| --- | --- | --- | --- | --- | --- |
|  | | Frequency | Percent | Valid Percent | Cumulative Percent |
| Valid | Poor Practice (<50% ) | 3 | 6.0 | 6.0 | 6.0 |
|  | Adequate Practice (50 - 75%) | 20 | 40.0 | 40.0 | 46.0 |
|  | Good Practice (>75%) | 27 | 54.0 | 54.0 | 100.0 |
|  | Total | 50 | 100.0 | 100.0 |  |

| **Designation_Code** | | | | | |
| --- | --- | --- | --- | --- | --- |
|  | | Frequency | Percent | Valid Percent | Cumulative Percent |
| Valid | Nursing staff | 17 | 34.0 | 34.0 | 34.0 |
|  | Doctors | 13 | 26.0 | 26.0 | 60.0 |
|  | Residents | 20 | 40.0 | 40.0 | 100.0 |
|  | Total | 50 | 100.0 | 100.0 |  |

| **List down the types of Hospital-acquired infection * List down the types of Hospital-acquired infection Crosstabulation** | | | | |
| --- | --- | --- | --- | --- |
| Count | | | | |
|  | | List down the types of Hospital-acquired infection | | Total |
|  |  | Wrong answer | Correct answer |  |
| List down the types of Hospital-acquired infection | Acinetobactor, VAP, e-coli, CAUTI, influenza, VRE, MRSA | 1 | 0 | 1 |
|  | CAUTI, pneumonia, CLABSI | 3 | 0 | 3 |
|  | CAUTI, SSI, sepsis, acinetobactor | 1 | 0 | 1 |
|  | CLABSI, VAP, SSI, CAUTI | 0 | 1 | 1 |
|  | E-coli, SSI, CAUTI, CLABSI, acinetobactor, pneumonia | 0 | 3 | 3 |
|  | Klebsila, acinetobactor, staph. | 1 | 0 | 1 |
|  | Klebsila, acinetobactor, MDR | 1 | 0 | 1 |
|  | Klebsila, CAUTI, SSI, staph, acinetobactor | 1 | 0 | 1 |
|  | MDR, CAUTI, acinetobactor, klebsila | 1 | 0 | 1 |
|  | MDR, MRSA, Acinetobactor, e-coli | 1 | 0 | 1 |
|  | MDR, MRSA,e-coli, kebsila, acinetobactor | 1 | 0 | 1 |
|  | MRSA, PTB, acinetobactor | 1 | 0 | 1 |
|  | Pneumonia | 1 | 0 | 1 |
|  | Pneumonia, CAUTI, sepsis, skin infection, CLABSI,SSI | 0 | 1 | 1 |
|  | Pneumonia, CAUTI, skin infection, MRSA, sepsis, bacteria | 2 | 0 | 2 |
|  | Pneumonia, SSI, CAUTI, CLABSI, skin | 0 | 1 | 1 |
|  | Pneumonia, SSI, CAUTI, VAP | 0 | 1 | 1 |
|  | Pneumonia, acinetobactor, klebsila | 1 | 0 | 1 |
|  | Pneumonia, bacteria | 3 | 0 | 3 |
|  | Pneumonia, CAUTI, MRSA, klebsila,SSI | 2 | 0 | 2 |
|  | SSI, CAUTI, CLABSI | 0 | 1 | 1 |
|  | SSI, medical devices | 1 | 0 | 1 |
|  | SSI, CAUTI, CLABSI, VAP | 0 | 1 | 1 |
|  | SSI, skin, CAUTI, VAP, | 0 | 1 | 1 |
|  | UTI, Central line | 1 | 0 | 1 |
|  | VAP, CAUTI, SSI, sepsis | 0 | 1 | 1 |
|  | VAP, CAUTI, acinetobactor, SSI, CLABSI | 0 | 4 | 4 |
|  | VAP, MDR, klebsila | 1 | 0 | 1 |
|  | VAP, CAUTI , SSI, clbsi | 0 | 1 | 1 |
|  | VAP, CAUTI, acinetobactor, klebsila | 1 | 0 | 1 |
|  | VAP, CAUTI, CLABSI, SSI | 0 | 1 | 1 |
|  | VAP, CAUTI, HAP | 3 | 0 | 3 |
|  | VAP, CAUTI, SSI, CLABSI | 0 | 2 | 2 |
|  | VAP, CLASBSI, SSI,CAUTI,BSI | 0 | 1 | 1 |
|  | VAP, SSI, CAUTI, CLABSI,BSI | 0 | 2 | 2 |
| Total | | 28 | 22 | 50 |

| **When do you think sodium hypochlorite solution is required to contain a blood spill? * When do you think sodium hypochlorite solution is required to contain a blood spill? Crosstabulation** | | | | |
| --- | --- | --- | --- | --- |
| Count | | | | |
|  | | When do you think sodium hypochlorite solution is required to contain a blood spill? | | Total |
|  |  | Wrong answer | Correct answer |  |
| When do you think sodium hypochlorite solution is required to contain a blood spill? | No idea | 17 | 0 | 17 |
|  | Spill < 10cms | 24 | 0 | 24 |
|  | Spill > 10cms | 0 | 1 | 1 |
|  | Spill > 15 cms | 0 | 8 | 8 |
| Total | | 41 | 9 | 50 |

| **How many workshops and seminars you have attended on HAI in the past two years : * How many workshops and seminars you have attended on HAI in the past two years : Crosstabulation** | | | | |
| --- | --- | --- | --- | --- |
| Count | | | | |
|  | | How many workshops and seminars you have attended on HAI in the past two years : | | Total |
|  |  | Wrong answer | Correct answer |  |
| How many workshops and seminars you have attended on HAI in the past two years : | None | 28 | 0 | 28 |
|  | 1 | 6 | 0 | 6 |
|  | 2 | 7 | 0 | 7 |
|  | 3 | 0 | 4 | 4 |
|  | 4 | 0 | 4 | 4 |
|  | 5 | 0 | 1 | 1 |
| Total | | 41 | 9 | 50 |

| **What in your opinion should be the frequency of these educational programs/ workshops and seminars on HAI ? * What in your opinion should be the frequency of these educational programs/ workshops and seminars on HAI ? Crosstabulation** | | | | |
| --- | --- | --- | --- | --- |
| Count | | | | |
|  | | What in your opinion should be the frequency of these educational programs/ workshops and seminars on HAI ? | | Total |
|  |  | Wrong answer | Correct answer |  |
| What in your opinion should be the frequency of these educational programs/ workshops and seminars on HAI ? | Annually | 0 | 13 | 13 |
|  | Every 3 months | 21 | 0 | 21 |
|  | every 6 months | 1 | 0 | 1 |
|  | Every 6 months | 15 | 0 | 15 |
| Total | | 37 | 13 | 50 |

| **How can you educate your fellow workers / Nursing staff and students * How can you educate your fellow workers / Nursing staff and students Crosstabulation** | | | | |
| --- | --- | --- | --- | --- |
| Count | | | | |
|  | | How can you educate your fellow workers / Nursing staff and students | | Total |
|  |  | Wrong answer | Correct answer |  |
| How can you educate your fellow workers / Nursing staff and students | Follow hand hygiene week | 3 | 0 | 3 |
|  | Lectures | 4 | 0 | 4 |
|  | Lectures, Follow hand hygiene week | 1 | 0 | 1 |
|  | Lectures, Orientation Programs, Follow hand hygiene week | 1 | 0 | 1 |
|  | Lectures, Workshops | 4 | 0 | 4 |
|  | Lectures, Workshops, Follow hand hygiene week | 1 | 0 | 1 |
|  | Lectures, Workshops, Orientation Programs | 0 | 2 | 2 |
|  | Lectures, Workshops, Orientation Programs, Follow hand hygiene week | 0 | 2 | 2 |
|  | Orientation Programs | 2 | 0 | 2 |
|  | Seminars , workshops | 2 | 0 | 2 |
|  | Seminars, Follow hand hygiene week | 1 | 0 | 1 |
|  | Seminars, Lectures, Workshops, Orientation Programs, Follow hand hygiene week | 0 | 1 | 1 |
|  | Seminars, Workshops | 1 | 0 | 1 |
|  | Seminars, Workshops, Orientation Programs | 4 | 0 | 4 |
|  | Seminars, Workshops, Orientation Programs, Follow hand hygiene week | 1 | 0 | 1 |
|  | Workshops | 4 | 0 | 4 |
|  | Workshops, Follow hand hygiene week | 3 | 0 | 3 |
|  | Workshops, Orientation Programs | 8 | 0 | 8 |
|  | You set an example, Lectures, Follow hand hygiene week | 1 | 0 | 1 |
|  | You set an example, Orientation Programs | 1 | 0 | 1 |
|  | You set an example, Seminars, Lectures, Workshops | 1 | 0 | 1 |
|  | You set an example, Seminars, Lectures, Workshops, Orientation Programs, Follow hand hygiene week | 0 | 1 | 1 |
|  | You set an example, Workshops, Follow hand hygiene week | 1 | 0 | 1 |
| Total | | 44 | 6 | 50 |

| **Who is in charge of managing blood spill and body fluid spill? * Who is in charge of managing blood spill and body fluid spill? Crosstabulation** | | | |
| --- | --- | --- | --- |
| Count | | | |
|  | | Who is in charge of managing blood spill and body fluid spill? | Total |
|  |  | Correct answer |  |
| Who is in charge of managing blood spill and body fluid spill? | Consultant/specialists | 1 | 1 |
|  | Consultant/specialists, Nurses | 1 | 1 |
|  | Consultant/specialists, Residents, Nurses, Interns, Health worker | 1 | 1 |
|  | Health worker | 14 | 14 |
|  | Interns | 1 | 1 |
|  | Nurses | 22 | 22 |
|  | Nurses, Health worker | 2 | 2 |
|  | Nurses, Interns | 1 | 1 |
|  | Nurses, Interns, Health worker | 1 | 1 |
|  | Residents | 1 | 1 |
|  | Residents, Health worker | 1 | 1 |
|  | Residents, Nurses | 3 | 3 |
|  | Residents, Nurses, Health worker | 1 | 1 |
| Total | | 50 | 50 |

| **How many times during the day do you practice Hand hygiene? * How many times during the day do you practice Hand hygiene? Crosstabulation** | | | | |
| --- | --- | --- | --- | --- |
| Count | | | | |
|  | | How many times during the day do you practice Hand hygiene? | | Total |
|  |  | Wrong answer | Correct answer |  |
| How many times during the day do you practice Hand hygiene? | >3 | 0 | 2 | 2 |
|  | 10. | 0 | 13 | 13 |
|  | 15. | 0 | 4 | 4 |
|  | 2. | 1 | 0 | 1 |
|  | 20. | 0 | 3 | 3 |
|  | 3-4 times | 1 | 0 | 1 |
|  | 3. | 4 | 0 | 4 |
|  | 4. | 2 | 0 | 2 |
|  | 5-10 | 1 | 0 | 1 |
|  | 5. | 8 | 0 | 8 |
|  | 7. | 1 | 0 | 1 |
|  | 8. | 3 | 0 | 3 |
|  | After every patient | 0 | 2 | 2 |
|  | Depends on patient recieved | 0 | 1 | 1 |
|  | Many | 0 | 4 | 4 |
| Total | | 21 | 29 | 50 |

| **What do you use to decontaminate your hands? * What do you use to decontaminate your hands? Crosstabulation** | | | | |
| --- | --- | --- | --- | --- |
| Count | | | | |
|  | | What do you use to decontaminate your hands? | | Total |
|  |  | Wrong answer | Correct answer |  |
| What do you use to decontaminate your hands? | Alcohol Rub | 23 | 0 | 23 |
|  | Soap | 9 | 0 | 9 |
|  | Soap, Alcohol Rub | 0 | 18 | 18 |
| Total | | 32 | 18 | 50 |

| **How long do you wash your hands with soap and water? * How long do you wash your hands with soap and water? Crosstabulation** | | | | |
| --- | --- | --- | --- | --- |
| Count | | | | |
|  | | How long do you wash your hands with soap and water? | | Total |
|  |  | Wrong answer | Correct answer |  |
| How long do you wash your hands with soap and water? | 1 min | 0 | 24 | 24 |
|  | 10 sec | 2 | 0 | 2 |
|  | 2 mins | 0 | 5 | 5 |
|  | 20-30 secs | 0 | 5 | 5 |
|  | 25 secs | 0 | 1 | 1 |
|  | 3 mins | 0 | 1 | 1 |
|  | 30 secs | 0 | 1 | 1 |
|  | 30-60 secs | 0 | 2 | 2 |
|  | 40 secs | 0 | 1 | 1 |
|  | 40-60 secs | 0 | 4 | 4 |
|  | 40-80 secs | 0 | 1 | 1 |
|  | 45sec-1 min | 0 | 1 | 1 |
|  | 8 mins | 0 | 1 | 1 |
|  | less than 20 secs | 1 | 0 | 1 |
| Total | | 3 | 47 | 50 |

| **How long do you take to decontaminate your hands with alcohol rub? * How long do you take to decontaminate your hands with alcohol rub? Crosstabulation** | | | | |
| --- | --- | --- | --- | --- |
| Count | | | | |
|  | | How long do you take to decontaminate your hands with alcohol rub? | | Total |
|  |  | Wrong answer | Correct answer |  |
| How long do you take to decontaminate your hands with alcohol rub? | 1 min | 0 | 7 | 7 |
|  | 10 sec | 1 | 0 | 1 |
|  | 10-20 secs | 1 | 0 | 1 |
|  | 15 secs | 1 | 0 | 1 |
|  | 15-20 secs | 1 | 0 | 1 |
|  | 20 secs | 0 | 1 | 1 |
|  | 20-30 secs | 0 | 11 | 11 |
|  | 3 mins | 0 | 1 | 1 |
|  | 30 secs | 0 | 15 | 15 |
|  | 40 secs | 0 | 1 | 1 |
|  | 5 mins | 0 | 1 | 1 |
|  | 5 secs | 8 | 0 | 8 |
|  | 5-10secs | 1 | 0 | 1 |
| Total | | 13 | 37 | 50 |

| **Age: * Age_Grp Crosstabulation** | | | | | |
| --- | --- | --- | --- | --- | --- |
| Count | | | | | |
|  | | Age_Grp | | | Total |
|  |  | 20 - 29 yrs | 30 - 39 yrs | >= 40 yrs |  |
| Age: | 24 | 1 | 0 | 0 | 1 |
|  | 25 | 5 | 0 | 0 | 5 |
|  | 26 | 7 | 0 | 0 | 7 |
|  | 27 | 3 | 0 | 0 | 3 |
|  | 28 | 4 | 0 | 0 | 4 |
|  | 29 | 4 | 0 | 0 | 4 |
|  | 30 | 0 | 7 | 0 | 7 |
|  | 31 | 0 | 1 | 0 | 1 |
|  | 32 | 0 | 2 | 0 | 2 |
|  | 33 | 0 | 1 | 0 | 1 |
|  | 34 | 0 | 1 | 0 | 1 |
|  | 35 | 0 | 1 | 0 | 1 |
|  | 37 | 0 | 3 | 0 | 3 |
|  | 38 | 0 | 2 | 0 | 2 |
|  | 40 | 0 | 0 | 1 | 1 |
|  | 43 | 0 | 0 | 1 | 1 |
|  | 45 | 0 | 0 | 2 | 2 |
|  | 47 | 0 | 0 | 1 | 1 |
|  | 49 | 0 | 0 | 1 | 1 |
|  | 50 | 0 | 0 | 1 | 1 |
|  | 55 | 0 | 0 | 1 | 1 |
| Total | | 24 | 18 | 8 | 50 |

| **Know_Score * Know_Code Crosstabulation** | | | | |
| --- | --- | --- | --- | --- |
| Count | | | | |
|  | | Know_Code | | Total |
|  |  | Adequate Knowledge (50 - 75%) | Good knowledge (>75%) |  |
| Know_Score | 10 | 2 | 0 | 2 |
|  | 11 | 1 | 0 | 1 |
|  | 12 | 3 | 0 | 3 |
|  | 13 | 3 | 0 | 3 |
|  | 14 | 3 | 0 | 3 |
|  | 15 | 0 | 5 | 5 |
|  | 16 | 0 | 4 | 4 |
|  | 17 | 0 | 17 | 17 |
|  | 18 | 0 | 9 | 9 |
|  | 19 | 0 | 3 | 3 |
| Total | | 12 | 38 | 50 |

| **Atti_Score * Atti_Code Crosstabulation** | | | | |
| --- | --- | --- | --- | --- |
| Count | | | | |
|  | | Atti_Code | | Total |
|  |  | Adequate Attitude (50 - 75%) | Good Attitude (>75%) |  |
| Atti_Score | 5 | 2 | 0 | 2 |
|  | 6 | 25 | 0 | 25 |
|  | 7 | 12 | 0 | 12 |
|  | 8 | 0 | 11 | 11 |
| Total | | 39 | 11 | 50 |

| **Pract_Score * Pract_Code Crosstabulation** | | | | | |
| --- | --- | --- | --- | --- | --- |
| Count | | | | | |
|  | | Pract_Code | | | Total |
|  |  | Poor Practice (<50% ) | Adequate Practice (50 - 75%) | Good Practice (>75%) |  |
| Pract_Score | 3 | 3 | 0 | 0 | 3 |
|  | 4 | 0 | 6 | 0 | 6 |
|  | 5 | 0 | 7 | 0 | 7 |
|  | 6 | 0 | 7 | 0 | 7 |
|  | 7 | 0 | 0 | 18 | 18 |
|  | 8 | 0 | 0 | 9 | 9 |
| Total | | 3 | 20 | 27 | 50 |

| **Designation * Designation_Code Crosstabulation** | | | | | |
| --- | --- | --- | --- | --- | --- |
| Count | | | | | |
|  | | Designation_Code | | | Total |
|  |  | Nursing staff | Doctors | Residents |  |
| Designation | Anaesthetist | 0 | 2 | 0 | 2 |
|  | Cardiologist | 0 | 2 | 0 | 2 |
|  | Consultant | 0 | 4 | 0 | 4 |
|  | GP | 0 | 1 | 0 | 1 |
|  | IPC Nurse practitioner | 3 | 0 | 0 | 3 |
|  | Nurse | 13 | 0 | 0 | 13 |
|  | OR reception | 1 | 0 | 0 | 1 |
|  | Resident | 0 | 0 | 19 | 19 |
|  | Senior registar | 0 | 0 | 1 | 1 |
|  | Specialist | 0 | 4 | 0 | 4 |
| Total | | 17 | 13 | 20 | 50 |

**Difference in scores between three departments**

| **Report** | | | | |
| --- | --- | --- | --- | --- |
| Department_Code | | Know_Score | Atti_Score | Pract_Score |
| Pediatric ward and infection control department | Mean | 15.10 | 6.48 | 5.69 |
|  | Std. Deviation | 2.677 | .871 | 1.417 |
|  | N | 29 | 29 | 29 |
| Intensive care unit | Mean | 17.25 | 6.83 | 7.42 |
|  | Std. Deviation | .622 | .835 | .515 |
|  | N | 12 | 12 | 12 |
| Anesthesia and operation theater/Emergency | Mean | 16.67 | 6.89 | 6.00 |
|  | Std. Deviation | 1.500 | .928 | 1.803 |
|  | N | 9 | 9 | 9 |
| Total | Mean | 15.90 | 6.64 | 6.16 |
|  | Std. Deviation | 2.341 | .875 | 1.503 |
|  | N | 50 | 50 | 50 |

**Kruskal-Wallis Test**

| **Ranks** | | | |
| --- | --- | --- | --- |
|  | Department_Code | N | Mean Rank |
| Know_Score | Pediatric ward and infection control department | 29 | 21.24 |
|  | Intensive care unit | 12 | 33.46 |
|  | Anesthesia and operation theater/Emergency | 9 | 28.61 |
|  | Total | 50 |  |
| Atti_Score | Pediatric ward and infection control department | 29 | 23.07 |
|  | Intensive care unit | 12 | 28.67 |
|  | Anesthesia and operation theater/Emergency | 9 | 29.11 |
|  | Total | 50 |  |
| Pract_Score | Pediatric ward and infection control department | 29 | 20.47 |
|  | Intensive care unit | 12 | 38.13 |
|  | Anesthesia and operation theater/Emergency | 9 | 24.89 |
|  | Total | 50 |  |

| **Test Statistics^a,b^** | | | |
| --- | --- | --- | --- |
|  | Know_Score | Atti_Score | Pract_Score |
| Chi-Square | 6.782 | 2.263 | 13.266 |
| df | 2 | 2 | 2 |
| Asymp. Sig. | .034 | .323 | .001 |
| a. Kruskal Wallis Test | | | |
| b. Grouping Variable: Department_Code | | | |

There is difference in knowledge score between these three departments

There is difference in Practice score between these three departments

There is difference in knowledge score between Intensive care unit & Pediatric ward and infection control department

There is difference in Practice score between Intensive care unit & Pediatric ward and infection control department

**Difference in scores between gender**

| **Report** | | | | |
| --- | --- | --- | --- | --- |
| Gender | | Know_Score | Atti_Score | Pract_Score |
| Female | Mean | 15.73 | 6.68 | 6.45 |
|  | Std. Deviation | 2.142 | .894 | 1.262 |
|  | N | 22 | 22 | 22 |
| Male | Mean | 16.04 | 6.61 | 5.93 |
|  | Std. Deviation | 2.516 | .875 | 1.654 |
|  | N | 28 | 28 | 28 |
| Total | Mean | 15.90 | 6.64 | 6.16 |
|  | Std. Deviation | 2.341 | .875 | 1.503 |
|  | N | 50 | 50 | 50 |


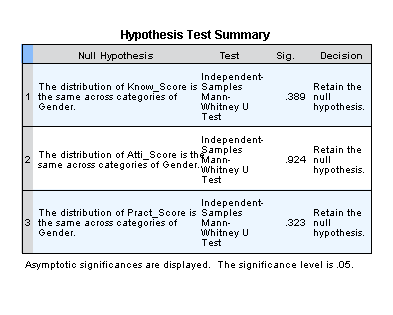


No differences between male and female in knowledge, attitude and practice

**Logistic Regression - Knowledge**

Age

| **Case Processing Summary** | | | |
| --- | --- | --- | --- |
| Unweighted Cases^a^ | | N | Percent |
| Selected Cases | Included in Analysis | 50 | 100.0 |
|  | Missing Cases | 0 | .0 |
|  | Total | 50 | 100.0 |
| Unselected Cases | | 0 | .0 |
| Total | | 50 | 100.0 |
| a. If weight is in effect, see classification table for the total number of cases. | | | |

| **Dependent Variable Encoding** | |
| --- | --- |
| Original Value | Internal Value |
| Adequate Knowledge (50 - 75%) | 0 |
| Good knowledge (>75%) | 1 |

**Block 0: Beginning Block**

| **Classification Table^a,b^** | | | | | |
| --- | --- | --- | --- | --- | --- |
|  | Observed | | Predicted | | |
|  |  |  | Know_Code | | Percentage Correct |
|  |  |  | Adequate Knowledge (50 - 75%) | Good knowledge (>75%) |  |
| Step 0 | Know_Code | Adequate Knowledge (50 - 75%) | 0 | 12 | .0 |
|  |  | Good knowledge (>75%) | 0 | 38 | 100.0 |
|  | Overall Percentage | |  |  | 76.0 |
| a. Constant is included in the model. | | | | | |
| b. The cut value is .500 | | | | | |

| **Variables in the Equation** | | | | | | | |
| --- | --- | --- | --- | --- | --- | --- | --- |
|  | | B | S.E. | Wald | df | Sig. | Exp(B) |
| Step 0 | Constant | 1.153 | .331 | 12.117 | 1 | .000 | 3.167 |

| **Variables not in the Equation** | | | | | |
| --- | --- | --- | --- | --- | --- |
|  | | | Score | df | Sig. |
| Step 0 | Variables | Age | .494 | 1 | .482 |
|  | Overall Statistics | | .494 | 1 | .482 |

**Block 1: Method = Enter**

| **Omnibus Tests of Model Coefficients** | | | | |
| --- | --- | --- | --- | --- |
|  | | Chi-square | df | Sig. |
| Step 1 | Step | .526 | 1 | .468 |
|  | Block | .526 | 1 | .468 |
|  | Model | .526 | 1 | .468 |

| **Model Summary** | | | |
| --- | --- | --- | --- |
| Step | -2 Log likelihood | Cox & Snell R Square | Nagelkerke R Square |
| 1 | 54.582^a^ | .010 | .016 |
| a. Estimation terminated at iteration number 4 because parameter estimates changed by less than .001. | | | |

| **Classification Table^a^** | | | | | |
| --- | --- | --- | --- | --- | --- |
|  | Observed | | Predicted | | |
|  |  |  | Know_Code | | Percentage Correct |
|  |  |  | Adequate Knowledge (50 - 75%) | Good knowledge (>75%) |  |
| Step 1 | Know_Code | Adequate Knowledge (50 - 75%) | 0 | 12 | .0 |
|  |  | Good knowledge (>75%) | 0 | 38 | 100.0 |
|  | Overall Percentage | |  |  | 76.0 |
| a. The cut value is .500 | | | | | |

| **Variables in the Equation** | | | | | | | | | |
| --- | --- | --- | --- | --- | --- | --- | --- | --- | --- |
|  | | B | S.E. | Wald | df | Sig. | Exp(B) | 95% C.I.for EXP(B) | |
|  |  |  |  |  |  |  |  | Lower | Upper |
| Step 1^a^ | Age | .034 | .049 | .484 | 1 | .486 | 1.035 | .940 | 1.140 |
|  | Constant | .066 | 1.572 | .002 | 1 | .966 | 1.068 |  |  |
| a. Variable(s) entered on step 1: Age. | | | | | | | | | |

Age - not significant

Gender

| **Case Processing Summary** | | | |
| --- | --- | --- | --- |
| Unweighted Cases^a^ | | N | Percent |
| Selected Cases | Included in Analysis | 50 | 100.0 |
|  | Missing Cases | 0 | .0 |
|  | Total | 50 | 100.0 |
| Unselected Cases | | 0 | .0 |
| Total | | 50 | 100.0 |
| a. If weight is in effect, see classification table for the total number of cases. | | | |

| **Dependent Variable Encoding** | |
| --- | --- |
| Original Value | Internal Value |
| Adequate Knowledge (50 - 75%) | 0 |
| Good knowledge (>75%) | 1 |

| **Categorical Variables Codings** | | | |
| --- | --- | --- | --- |
|  | | Frequency | Parameter coding |
|  |  |  | (1) |
| Gender | Female | 22 | 1.000 |
|  | Male | 28 | .000 |

**Block 0: Beginning Block**

| **Classification Table^a,b^** | | | | | |
| --- | --- | --- | --- | --- | --- |
|  | Observed | | Predicted | | |
|  |  |  | Know_Code | | Percentage Correct |
|  |  |  | Adequate Knowledge (50 - 75%) | Good knowledge (>75%) |  |
| Step 0 | Know_Code | Adequate Knowledge (50 - 75%) | 0 | 12 | .0 |
|  |  | Good knowledge (>75%) | 0 | 38 | 100.0 |
|  | Overall Percentage | |  |  | 76.0 |
| a. Constant is included in the model. | | | | | |
| b. The cut value is .500 | | | | | |

| **Variables in the Equation** | | | | | | | |
| --- | --- | --- | --- | --- | --- | --- | --- |
|  | | B | S.E. | Wald | df | Sig. | Exp(B) |
| Step 0 | Constant | 1.153 | .331 | 12.117 | 1 | .000 | 3.167 |

| **Variables not in the Equation** | | | | | |
| --- | --- | --- | --- | --- | --- |
|  | | | Score | df | Sig. |
| Step 0 | Variables | Gender(1) | .231 | 1 | .631 |
|  | Overall Statistics | | .231 | 1 | .631 |

**Block 1: Method = Enter**

| **Omnibus Tests of Model Coefficients** | | | | |
| --- | --- | --- | --- | --- |
|  | | Chi-square | df | Sig. |
| Step 1 | Step | .230 | 1 | .632 |
|  | Block | .230 | 1 | .632 |
|  | Model | .230 | 1 | .632 |

| **Model Summary** | | | |
| --- | --- | --- | --- |
| Step | -2 Log likelihood | Cox & Snell R Square | Nagelkerke R Square |
| 1 | 54.878^a^ | .005 | .007 |
| a. Estimation terminated at iteration number 4 because parameter estimates changed by less than .001. | | | |

| **Classification Table^a^** | | | | | |
| --- | --- | --- | --- | --- | --- |
|  | Observed | | Predicted | | |
|  |  |  | Know_Code | | Percentage Correct |
|  |  |  | Adequate Knowledge (50 - 75%) | Good knowledge (>75%) |  |
| Step 1 | Know_Code | Adequate Knowledge (50 - 75%) | 0 | 12 | .0 |
|  |  | Good knowledge (>75%) | 0 | 38 | 100.0 |
|  | Overall Percentage | |  |  | 76.0 |
| a. The cut value is .500 | | | | | |

| **Variables in the Equation** | | | | | | | | | |
| --- | --- | --- | --- | --- | --- | --- | --- | --- | --- |
|  | | B | S.E. | Wald | df | Sig. | Exp(B) | 95% C.I.for EXP(B) | |
|  |  |  |  |  |  |  |  | Lower | Upper |
| Step 1^a^ | Gender(1) | -.318 | .664 | .230 | 1 | .632 | .727 | .198 | 2.674 |
|  | Constant | 1.299 | .461 | 7.958 | 1 | .005 | 3.667 |  |  |
| a. Variable(s) entered on step 1: Gender. | | | | | | | | | |

Gender- not significant

Department

| **Case Processing Summary** | | | |
| --- | --- | --- | --- |
| Unweighted Cases^a^ | | N | Percent |
| Selected Cases | Included in Analysis | 50 | 100.0 |
|  | Missing Cases | 0 | .0 |
|  | Total | 50 | 100.0 |
| Unselected Cases | | 0 | .0 |
| Total | | 50 | 100.0 |
| a. If weight is in effect, see classification table for the total number of cases. | | | |

| **Dependent Variable Encoding** | |
| --- | --- |
| Original Value | Internal Value |
| Adequate Knowledge (50 - 75%) | 0 |
| Good knowledge (>75%) | 1 |

| **Categorical Variables Codings** | | | | |
| --- | --- | --- | --- | --- |
|  | | Frequency | Parameter coding | |
|  |  |  | (1) | (2) |
| Department_Code | Pediatric ward and infection control department | 29 | 1.000 | .000 |
|  | Intensive care unit | 12 | .000 | 1.000 |
|  | Anesthesia and operation theater/Emergency | 9 | .000 | .000 |

**Block 0: Beginning Block**

| **Classification Table^a,b^** | | | | | |
| --- | --- | --- | --- | --- | --- |
|  | Observed | | Predicted | | |
|  |  |  | Know_Code | | Percentage Correct |
|  |  |  | Adequate Knowledge (50 - 75%) | Good knowledge (>75%) |  |
| Step 0 | Know_Code | Adequate Knowledge (50 - 75%) | 0 | 12 | .0 |
|  |  | Good knowledge (>75%) | 0 | 38 | 100.0 |
|  | Overall Percentage | |  |  | 76.0 |
| a. Constant is included in the model. | | | | | |
| b. The cut value is .500 | | | | | |

| **Variables in the Equation** | | | | | | | |
| --- | --- | --- | --- | --- | --- | --- | --- |
|  | | B | S.E. | Wald | df | Sig. | Exp(B) |
| Step 0 | Constant | 1.153 | .331 | 12.117 | 1 | .000 | 3.167 |

| **Variables not in the Equation** | | | | | |
| --- | --- | --- | --- | --- | --- |
|  | | | Score | df | Sig. |
| Step 0 | Variables | Department_Code | 7.695 | 2 | .021 |
|  |  | Department_Code(1) | 7.347 | 1 | .007 |
|  |  | Department_Code(2) | 4.986 | 1 | .026 |
|  | Overall Statistics | | 7.695 | 2 | .021 |

**Block 1: Method = Enter**

| **Omnibus Tests of Model Coefficients** | | | | |
| --- | --- | --- | --- | --- |
|  | | Chi-square | df | Sig. |
| Step 1 | Step | 10.333 | 2 | .006 |
|  | Block | 10.333 | 2 | .006 |
|  | Model | 10.333 | 2 | .006 |

| **Model Summary** | | | |
| --- | --- | --- | --- |
| Step | -2 Log likelihood | Cox & Snell R Square | Nagelkerke R Square |
| 1 | 44.775^a^ | .187 | .280 |
| a. Estimation terminated at iteration number 20 because maximum iterations has been reached. Final solution cannot be found. | | | |

| **Classification Table^a^** | | | | | |
| --- | --- | --- | --- | --- | --- |
|  | Observed | | Predicted | | |
|  |  |  | Know_Code | | Percentage Correct |
|  |  |  | Adequate Knowledge (50 - 75%) | Good knowledge (>75%) |  |
| Step 1 | Know_Code | Adequate Knowledge (50 - 75%) | 0 | 12 | .0 |
|  |  | Good knowledge (>75%) | 0 | 38 | 100.0 |
|  | Overall Percentage | |  |  | 76.0 |
| a. The cut value is .500 | | | | | |

| **Variables in the Equation** | | | | | | | | | |
| --- | --- | --- | --- | --- | --- | --- | --- | --- | --- |
|  | | B | S.E. | Wald | df | Sig. | Exp(B) | 95% C.I.for EXP(B) | |
|  |  |  |  |  |  |  |  | Lower | Upper |
| Step 1^a^ | Department_Code |  |  | 1.981 | 2 | .371 |  |  |  |
|  | Department_Code(1) | -1.587 | 1.128 | 1.981 | 1 | .159 | .205 | .022 | 1.865 |
|  | Department_Code(2) | 19.123 | 11602.711 | .000 | 1 | .999 | 201934358.037 | .000 | . |
|  | Constant | 2.079 | 1.061 | 3.844 | 1 | .050 | 8.000 |  |  |
| a. Variable(s) entered on step 1: Department_Code. | | | | | | | | | |

Department not significant

| **Department_Code * Know_Code Crosstabulation** | | | | |
| --- | --- | --- | --- | --- |
| Count | | | | |
|  | | Know_Code | | Total |
|  |  | Adequate Knowledge (50 - 75%) | Good knowledge (>75%) |  |
| Department_Code | Pediatric ward and infection control department | 11 | 18 | 29 |
|  | Intensive care unit | 0 | 12 | 12 |
|  | Anesthesia and operation theate/Emergencyr | 1 | 8 | 9 |
| Total | | 12 | 38 | 50 |

Designation

| **Case Processing Summary** | | | |
| --- | --- | --- | --- |
| Unweighted Cases^a^ | | N | Percent |
| Selected Cases | Included in Analysis | 50 | 100.0 |
|  | Missing Cases | 0 | .0 |
|  | Total | 50 | 100.0 |
| Unselected Cases | | 0 | .0 |
| Total | | 50 | 100.0 |
| a. If weight is in effect, see classification table for the total number of cases. | | | |

| **Dependent Variable Encoding** | |
| --- | --- |
| Original Value | Internal Value |
| Adequate Knowledge (50 - 75%) | 0 |
| Good knowledge (>75%) | 1 |

| **Categorical Variables Codings** | | | | |
| --- | --- | --- | --- | --- |
|  | | Frequency | Parameter coding | |
|  |  |  | (1) | (2) |
| Designation_Code | Nursing staff | 17 | 1.000 | .000 |
|  | Doctors | 13 | .000 | 1.000 |
|  | Residents | 20 | .000 | .000 |

**Block 0: Beginning Block**

| **Classification Table^a,b^** | | | | | |
| --- | --- | --- | --- | --- | --- |
|  | Observed | | Predicted | | |
|  |  |  | Know_Code | | Percentage Correct |
|  |  |  | Adequate Knowledge (50 - 75%) | Good knowledge (>75%) |  |
| Step 0 | Know_Code | Adequate Knowledge (50 - 75%) | 0 | 12 | .0 |
|  |  | Good knowledge (>75%) | 0 | 38 | 100.0 |
|  | Overall Percentage | |  |  | 76.0 |
| a. Constant is included in the model. | | | | | |
| b. The cut value is .500 | | | | | |

| **Variables in the Equation** | | | | | | | |
| --- | --- | --- | --- | --- | --- | --- | --- |
|  | | B | S.E. | Wald | df | Sig. | Exp(B) |
| Step 0 | Constant | 1.153 | .331 | 12.117 | 1 | .000 | 3.167 |

| **Variables not in the Equation** | | | | | |
| --- | --- | --- | --- | --- | --- |
|  | | | Score | df | Sig. |
| Step 0 | Variables | Designation_Code | 2.728 | 2 | .256 |
|  |  | Designation_Code(1) | 2.114 | 1 | .146 |
|  |  | Designation_Code(2) | .008 | 1 | .928 |
|  | Overall Statistics | | 2.728 | 2 | .256 |

**Block 1: Method = Enter**

| **Omnibus Tests of Model Coefficients** | | | | |
| --- | --- | --- | --- | --- |
|  | | Chi-square | df | Sig. |
| Step 1 | Step | 2.850 | 2 | .241 |
|  | Block | 2.850 | 2 | .241 |
|  | Model | 2.850 | 2 | .241 |

| **Model Summary** | | | |
| --- | --- | --- | --- |
| Step | -2 Log likelihood | Cox & Snell R Square | Nagelkerke R Square |
| 1 | 52.258^a^ | .055 | .083 |
| a. Estimation terminated at iteration number 5 because parameter estimates changed by less than .001. | | | |

| **Classification Table^a^** | | | | | |
| --- | --- | --- | --- | --- | --- |
|  | Observed | | Predicted | | |
|  |  |  | Know_Code | | Percentage Correct |
|  |  |  | Adequate Knowledge (50 - 75%) | Good knowledge (>75%) |  |
| Step 1 | Know_Code | Adequate Knowledge (50 - 75%) | 0 | 12 | .0 |
|  |  | Good knowledge (>75%) | 0 | 38 | 100.0 |
|  | Overall Percentage | |  |  | 76.0 |
| a. The cut value is .500 | | | | | |

| **Variables in the Equation** | | | | | | | | | |
| --- | --- | --- | --- | --- | --- | --- | --- | --- | --- |
|  | | B | S.E. | Wald | df | Sig. | Exp(B) | 95% C.I.for EXP(B) | |
|  |  |  |  |  |  |  |  | Lower | Upper |
| Step 1^a^ | Designation_Code |  |  | 2.542 | 2 | .281 |  |  |  |
|  | Designation_Code(1) | 1.396 | .887 | 2.478 | 1 | .115 | 4.038 | .710 | 22.966 |
|  | Designation_Code(2) | .585 | .808 | .524 | 1 | .469 | 1.795 | .368 | 8.749 |
|  | Constant | .619 | .469 | 1.744 | 1 | .187 | 1.857 |  |  |
| a. Variable(s) entered on step 1: Designation_Code. | | | | | | | | | |

Designation - not significant

******************************************************************************************************

**Logistic Regression – Attitude**

Age

| **Case Processing Summary** | | | |
| --- | --- | --- | --- |
| Unweighted Cases^a^ | | N | Percent |
| Selected Cases | Included in Analysis | 50 | 100.0 |
|  | Missing Cases | 0 | .0 |
|  | Total | 50 | 100.0 |
| Unselected Cases | | 0 | .0 |
| Total | | 50 | 100.0 |
| a. If weight is in effect, see classification table for the total number of cases. | | | |

| **Dependent Variable Encoding** | |
| --- | --- |
| Original Value | Internal Value |
| Adequate Attitude (50 - 75%) | 0 |
| Good Attitude (>75%) | 1 |

**Block 0: Beginning Block**

| **Classification Table^a,b^** | | | | | |
| --- | --- | --- | --- | --- | --- |
|  | Observed | | Predicted | | |
|  |  |  | Atti_Code | | Percentage Correct |
|  |  |  | Adequate Attitude (50 - 75%) | Good Attitude (>75%) |  |
| Step 0 | Atti_Code | Adequate Attitude (50 - 75%) | 39 | 0 | 100.0 |
|  |  | Good Attitude (>75%) | 11 | 0 | .0 |
|  | Overall Percentage | |  |  | 78.0 |
| a. Constant is included in the model. | | | | | |
| b. The cut value is .500 | | | | | |

| **Variables in the Equation** | | | | | | | |
| --- | --- | --- | --- | --- | --- | --- | --- |
|  | | B | S.E. | Wald | df | Sig. | Exp(B) |
| Step 0 | Constant | -1.266 | .341 | 13.744 | 1 | .000 | .282 |

| **Variables not in the Equation** | | | | | |
| --- | --- | --- | --- | --- | --- |
|  | | | Score | df | Sig. |
| Step 0 | Variables | Age | .262 | 1 | .609 |
|  | Overall Statistics | | .262 | 1 | .609 |

**Block 1: Method = Enter**

| **Omnibus Tests of Model Coefficients** | | | | |
| --- | --- | --- | --- | --- |
|  | | Chi-square | df | Sig. |
| Step 1 | Step | .252 | 1 | .616 |
|  | Block | .252 | 1 | .616 |
|  | Model | .252 | 1 | .616 |

| **Model Summary** | | | |
| --- | --- | --- | --- |
| Step | -2 Log likelihood | Cox & Snell R Square | Nagelkerke R Square |
| 1 | 52.439^a^ | .005 | .008 |
| a. Estimation terminated at iteration number 4 because parameter estimates changed by less than .001. | | | |

| **Classification Table^a^** | | | | | |
| --- | --- | --- | --- | --- | --- |
|  | Observed | | Predicted | | |
|  |  |  | Atti_Code | | Percentage Correct |
|  |  |  | Adequate Attitude (50 - 75%) | Good Attitude (>75%) |  |
| Step 1 | Atti_Code | Adequate Attitude (50 - 75%) | 39 | 0 | 100.0 |
|  |  | Good Attitude (>75%) | 11 | 0 | .0 |
|  | Overall Percentage | |  |  | 78.0 |
| a. The cut value is .500 | | | | | |

| **Variables in the Equation** | | | | | | | | | |
| --- | --- | --- | --- | --- | --- | --- | --- | --- | --- |
|  | | B | S.E. | Wald | df | Sig. | Exp(B) | 95% C.I.for EXP(B) | |
|  |  |  |  |  |  |  |  | Lower | Upper |
| Step 1^a^ | Age | .022 | .043 | .259 | 1 | .611 | 1.022 | .939 | 1.113 |
|  | Constant | -1.983 | 1.464 | 1.835 | 1 | .176 | .138 |  |  |
| a. Variable(s) entered on step 1: Age. | | | | | | | | | |

Age - not significant

Gender

| **Case Processing Summary** | | | |
| --- | --- | --- | --- |
| Unweighted Cases^a^ | | N | Percent |
| Selected Cases | Included in Analysis | 50 | 100.0 |
|  | Missing Cases | 0 | .0 |
|  | Total | 50 | 100.0 |
| Unselected Cases | | 0 | .0 |
| Total | | 50 | 100.0 |
| a. If weight is in effect, see classification table for the total number of cases. | | | |

| **Dependent Variable Encoding** | |
| --- | --- |
| Original Value | Internal Value |
| Adequate Attitude (50 - 75%) | 0 |
| Good Attitude (>75%) | 1 |

| **Categorical Variables Codings** | | | |
| --- | --- | --- | --- |
|  | | Frequency | Parameter coding |
|  |  |  | (1) |
| Gender | Female | 22 | 1.000 |
|  | Male | 28 | .000 |

**Block 0: Beginning Block**

| **Classification Table^a,b^** | | | | | |
| --- | --- | --- | --- | --- | --- |
|  | Observed | | Predicted | | |
|  |  |  | Atti_Code | | Percentage Correct |
|  |  |  | Adequate Attitude (50 - 75%) | Good Attitude (>75%) |  |
| Step 0 | Atti_Code | Adequate Attitude (50 - 75%) | 39 | 0 | 100.0 |
|  |  | Good Attitude (>75%) | 11 | 0 | .0 |
|  | Overall Percentage | |  |  | 78.0 |
| a. Constant is included in the model. | | | | | |
| b. The cut value is .500 | | | | | |

| **Variables in the Equation** | | | | | | | |
| --- | --- | --- | --- | --- | --- | --- | --- |
|  | | B | S.E. | Wald | df | Sig. | Exp(B) |
| Step 0 | Constant | -1.266 | .341 | 13.744 | 1 | .000 | .282 |

| **Variables not in the Equation** | | | | | |
| --- | --- | --- | --- | --- | --- |
|  | | | Score | df | Sig. |
| Step 0 | Variables | Gender(1) | .636 | 1 | .425 |
|  | Overall Statistics | | .636 | 1 | .425 |

**Block 1: Method = Enter**

| **Omnibus Tests of Model Coefficients** | | | | |
| --- | --- | --- | --- | --- |
|  | | Chi-square | df | Sig. |
| Step 1 | Step | .633 | 1 | .426 |
|  | Block | .633 | 1 | .426 |
|  | Model | .633 | 1 | .426 |

| **Model Summary** | | | |
| --- | --- | --- | --- |
| Step | -2 Log likelihood | Cox & Snell R Square | Nagelkerke R Square |
| 1 | 52.058^a^ | .013 | .019 |
| a. Estimation terminated at iteration number 4 because parameter estimates changed by less than .001. | | | |

| **Classification Table^a^** | | | | | |
| --- | --- | --- | --- | --- | --- |
|  | Observed | | Predicted | | |
|  |  |  | Atti_Code | | Percentage Correct |
|  |  |  | Adequate Attitude (50 - 75%) | Good Attitude (>75%) |  |
| Step 1 | Atti_Code | Adequate Attitude (50 - 75%) | 39 | 0 | 100.0 |
|  |  | Good Attitude (>75%) | 11 | 0 | .0 |
|  | Overall Percentage | |  |  | 78.0 |
| a. The cut value is .500 | | | | | |

| **Variables in the Equation** | | | | | | | | | |
| --- | --- | --- | --- | --- | --- | --- | --- | --- | --- |
|  | | B | S.E. | Wald | df | Sig. | Exp(B) | 95% C.I.for EXP(B) | |
|  |  |  |  |  |  |  |  | Lower | Upper |
| Step 1^a^ | Gender(1) | .545 | .687 | .629 | 1 | .428 | 1.725 | .448 | 6.637 |
|  | Constant | -1.526 | .493 | 9.565 | 1 | .002 | .217 |  |  |
| a. Variable(s) entered on step 1: Gender. | | | | | | | | | |

Gender - not significant

Department

| **Case Processing Summary** | | | |
| --- | --- | --- | --- |
| Unweighted Cases^a^ | | N | Percent |
| Selected Cases | Included in Analysis | 50 | 100.0 |
|  | Missing Cases | 0 | .0 |
|  | Total | 50 | 100.0 |
| Unselected Cases | | 0 | .0 |
| Total | | 50 | 100.0 |
| a. If weight is in effect, see classification table for the total number of cases. | | | |

| **Dependent Variable Encoding** | |
| --- | --- |
| Original Value | Internal Value |
| Adequate Attitude (50 - 75%) | 0 |
| Good Attitude (>75%) | 1 |

| **Categorical Variables Codings** | | | | |
| --- | --- | --- | --- | --- |
|  | | Frequency | Parameter coding | |
|  |  |  | (1) | (2) |
| Department_Code | Pediatric ward and infection control department | 29 | 1.000 | .000 |
|  | Intensive care unit | 12 | .000 | 1.000 |
|  | Anesthesia and operation theater | 9 | .000 | .000 |

**Block 0: Beginning Block**

| **Classification Table^a,b^** | | | | | |
| --- | --- | --- | --- | --- | --- |
|  | Observed | | Predicted | | |
|  |  |  | Atti_Code | | Percentage Correct |
|  |  |  | Adequate Attitude (50 - 75%) | Good Attitude (>75%) |  |
| Step 0 | Atti_Code | Adequate Attitude (50 - 75%) | 39 | 0 | 100.0 |
|  |  | Good Attitude (>75%) | 11 | 0 | .0 |
|  | Overall Percentage | |  |  | 78.0 |
| a. Constant is included in the model. | | | | | |
| b. The cut value is .500 | | | | | |

| **Variables in the Equation** | | | | | | | |
| --- | --- | --- | --- | --- | --- | --- | --- |
|  | | B | S.E. | Wald | df | Sig. | Exp(B) |
| Step 0 | Constant | -1.266 | .341 | 13.744 | 1 | .000 | .282 |

| **Variables not in the Equation** | | | | | |
| --- | --- | --- | --- | --- | --- |
|  | | | Score | df | Sig. |
| Step 0 | Variables | Department_Code | 1.119 | 2 | .571 |
|  |  | Department_Code(1) | .911 | 1 | .340 |
|  |  | Department_Code(2) | .083 | 1 | .774 |
|  | Overall Statistics | | 1.119 | 2 | .571 |

**Block 1: Method = Enter**

| **Omnibus Tests of Model Coefficients** | | | | |
| --- | --- | --- | --- | --- |
|  | | Chi-square | df | Sig. |
| Step 1 | Step | 1.075 | 2 | .584 |
|  | Block | 1.075 | 2 | .584 |
|  | Model | 1.075 | 2 | .584 |

| **Model Summary** | | | |
| --- | --- | --- | --- |
| Step | -2 Log likelihood | Cox & Snell R Square | Nagelkerke R Square |
| 1 | 51.615^a^ | .021 | .033 |
| a. Estimation terminated at iteration number 4 because parameter estimates changed by less than .001. | | | |

| **Classification Table^a^** | | | | | |
| --- | --- | --- | --- | --- | --- |
|  | Observed | | Predicted | | |
|  |  |  | Atti_Code | | Percentage Correct |
|  |  |  | Adequate Attitude (50 - 75%) | Good Attitude (>75%) |  |
| Step 1 | Atti_Code | Adequate Attitude (50 - 75%) | 39 | 0 | 100.0 |
|  |  | Good Attitude (>75%) | 11 | 0 | .0 |
|  | Overall Percentage | |  |  | 78.0 |
| a. The cut value is .500 | | | | | |

| **Variables in the Equation** | | | | | | | | | |
| --- | --- | --- | --- | --- | --- | --- | --- | --- | --- |
|  | | B | S.E. | Wald | df | Sig. | Exp(B) | 95% C.I.for EXP(B) | |
|  |  |  |  |  |  |  |  | Lower | Upper |
| Step 1^a^ | Department_Code |  |  | 1.090 | 2 | .580 |  |  |  |
|  | Department_Code(1) | -.875 | .861 | 1.033 | 1 | .309 | .417 | .077 | 2.253 |
|  | Department_Code(2) | -.405 | .972 | .174 | 1 | .677 | .667 | .099 | 4.478 |
|  | Constant | -.693 | .707 | .961 | 1 | .327 | .500 |  |  |
| a. Variable(s) entered on step 1: Department_Code. | | | | | | | | | |

Department- not significant

Designation

| **Case Processing Summary** | | | |
| --- | --- | --- | --- |
| Unweighted Cases^a^ | | N | Percent |
| Selected Cases | Included in Analysis | 50 | 100.0 |
|  | Missing Cases | 0 | .0 |
|  | Total | 50 | 100.0 |
| Unselected Cases | | 0 | .0 |
| Total | | 50 | 100.0 |
| a. If weight is in effect, see classification table for the total number of cases. | | | |

| **Dependent Variable Encoding** | |
| --- | --- |
| Original Value | Internal Value |
| Adequate Attitude (50 - 75%) | 0 |
| Good Attitude (>75%) | 1 |

| **Categorical Variables Codings** | | | | |
| --- | --- | --- | --- | --- |
|  | | Frequency | Parameter coding | |
|  |  |  | (1) | (2) |
| Designation_Code | Nursing staff | 17 | 1.000 | .000 |
|  | Doctors | 13 | .000 | 1.000 |
|  | Residents | 20 | .000 | .000 |

**Block 0: Beginning Block**

| **Classification Table^a,b^** | | | | | |
| --- | --- | --- | --- | --- | --- |
|  | Observed | | Predicted | | |
|  |  |  | Atti_Code | | Percentage Correct |
|  |  |  | Adequate Attitude (50 - 75%) | Good Attitude (>75%) |  |
| Step 0 | Atti_Code | Adequate Attitude (50 - 75%) | 39 | 0 | 100.0 |
|  |  | Good Attitude (>75%) | 11 | 0 | .0 |
|  | Overall Percentage | |  |  | 78.0 |
| a. Constant is included in the model. | | | | | |
| b. The cut value is .500 | | | | | |

| **Variables in the Equation** | | | | | | | |
| --- | --- | --- | --- | --- | --- | --- | --- |
|  | | B | S.E. | Wald | df | Sig. | Exp(B) |
| Step 0 | Constant | -1.266 | .341 | 13.744 | 1 | .000 | .282 |

| **Variables not in the Equation** | | | | | |
| --- | --- | --- | --- | --- | --- |
|  | | | Score | df | Sig. |
| Step 0 | Variables | Designation_Code | 3.438 | 2 | .179 |
|  |  | Designation_Code(1) | 2.653 | 1 | .103 |
|  |  | Designation_Code(2) | .012 | 1 | .913 |
|  | Overall Statistics | | 3.438 | 2 | .179 |

**Block 1: Method = Enter**

| **Omnibus Tests of Model Coefficients** | | | | |
| --- | --- | --- | --- | --- |
|  | | Chi-square | df | Sig. |
| Step 1 | Step | 3.568 | 2 | .168 |
|  | Block | 3.568 | 2 | .168 |
|  | Model | 3.568 | 2 | .168 |

| **Model Summary** | | | |
| --- | --- | --- | --- |
| Step | -2 Log likelihood | Cox & Snell R Square | Nagelkerke R Square |
| 1 | 49.123^a^ | .069 | .106 |
| a. Estimation terminated at iteration number 5 because parameter estimates changed by less than .001. | | | |

| **Classification Table^a^** | | | | | |
| --- | --- | --- | --- | --- | --- |
|  | Observed | | Predicted | | |
|  |  |  | Atti_Code | | Percentage Correct |
|  |  |  | Adequate Attitude (50 - 75%) | Good Attitude (>75%) |  |
| Step 1 | Atti_Code | Adequate Attitude (50 - 75%) | 39 | 0 | 100.0 |
|  |  | Good Attitude (>75%) | 11 | 0 | .0 |
|  | Overall Percentage | |  |  | 78.0 |
| a. The cut value is .500 | | | | | |

| **Variables in the Equation** | | | | | | | | | |
| --- | --- | --- | --- | --- | --- | --- | --- | --- | --- |
|  | | B | S.E. | Wald | df | Sig. | Exp(B) | 95% C.I.for EXP(B) | |
|  |  |  |  |  |  |  |  | Lower | Upper |
| Step 1^a^ | Designation_Code |  |  | 3.128 | 2 | .209 |  |  |  |
|  | Designation_Code(1) | 1.591 | .902 | 3.113 | 1 | .078 | 4.909 | .838 | 28.745 |
|  | Designation_Code(2) | .993 | .994 | .998 | 1 | .318 | 2.700 | .385 | 18.959 |
|  | Constant | -2.197 | .745 | 8.690 | 1 | .003 | .111 |  |  |
| a. Variable(s) entered on step 1: Designation_Code. | | | | | | | | | |

Designation_ not significant

******************************************************************************************************

**Logistic Regression – Attitude**

Not possible

**Note:**

**For knowledge & attitude logistic regression was done** Adequate Knowledge / Adequate Attitude has control AND Good knowledge / Good Attitude as case

Since we have three categories for Practice - logistic regression Not possible

| **Know_Code** | | | | | |
| --- | --- | --- | --- | --- | --- |
|  | | Frequency | Percent | Valid Percent | Cumulative Percent |
| Valid | Adequate Knowledge (50 - 75%) | 12 | 24.0 | 24.0 | 24.0 |
|  | Good knowledge (>75%) | 38 | 76.0 | 76.0 | 100.0 |
|  | Total | 50 | 100.0 | 100.0 |  |

| **Atti_Code** | | | | | |
| --- | --- | --- | --- | --- | --- |
|  | | Frequency | Percent | Valid Percent | Cumulative Percent |
| Valid | Adequate Attitude (50 - 75%) | 39 | 78.0 | 78.0 | 78.0 |
|  | Good Attitude (>75%) | 11 | 22.0 | 22.0 | 100.0 |
|  | Total | 50 | 100.0 | 100.0 |  |

| **Pract_Code** | | | | | |
| --- | --- | --- | --- | --- | --- |
|  | | Frequency | Percent | Valid Percent | Cumulative Percent |
| Valid | Poor Practice (<50% ) | 3 | 6.0 | 6.0 | 6.0 |
|  | Adequate Practice (50 - 75%) | 20 | 40.0 | 40.0 | 46.0 |
|  | Good Practice (>75%) | 27 | 54.0 | 54.0 | 100.0 |
|  | Total | 50 | 100.0 | 100.0 |  |
